# Ser/Thr Kinase-Dependent Phosphorylation of the Peptidoglycan Hydrolase CwlA Controls Its Export and Modulates Cell Division in Clostridioides difficile

**DOI:** 10.1128/mBio.00519-21

**Published:** 2021-05-18

**Authors:** Transito Garcia-Garcia, Sandrine Poncet, Elodie Cuenot, Thibaut Douché, Quentin Giai Gianetto, Johann Peltier, Pascal Courtin, Marie-Pierre Chapot-Chartier, Mariette Matondo, Bruno Dupuy, Thomas Candela, Isabelle Martin-Verstraete

**Affiliations:** a Laboratoire Pathogénese des Bactéries Anaérobies, Institut Pasteur, UMR CNRS 2001, Université de Paris, Paris, France; b Université Paris-Saclay, INRAE, AgroParisTech, Micalis Institute, Jouy-en-Josas, France; c Plateforme Protéomique, Unité de Technologie et Service Spectrométrie de Masse pour la Biologie, CNRS USR 2000, Institut Pasteur, Paris, France; d Hub de Bioinformatique et Statistique, Departement de Biologie Computationelle, USR3756, Institut Pasteur, Paris, France; e Université Paris-Saclay, CEA, CNRS, Institute for Integrative Biology of the Cells (I2BC), Gif-sur-Yvette, France; f Institut Universitaire de France, Paris, France; University of Delaware

**Keywords:** Hanks kinase, cell wall hydrolases, peptidoglycan, phosphorylation, control of exportation, envelope homeostasis, cell division, *Clostridium difficile*, anaerobes, cell wall, kinases, protein phosphorylation

## Abstract

Cell growth and division require a balance between synthesis and hydrolysis of the peptidoglycan (PG). Inhibition of PG synthesis or uncontrolled PG hydrolysis can be lethal for the cells, making it imperative to control peptidoglycan hydrolase (PGH) activity. The synthesis or activity of several key enzymes along the PG biosynthetic pathway is controlled by the Hanks-type serine/threonine kinases (STKs). In Gram-positive bacteria, inactivation of genes encoding STKs is associated with a range of phenotypes, including cell division defects and changes in cell wall metabolism, but only a few kinase substrates and associated mechanisms have been identified. We previously demonstrated that STK-PrkC plays an important role in cell division, cell wall metabolism, and resistance to antimicrobial compounds in the human enteropathogen *Clostridioides difficile*. In this work, we characterized a PG hydrolase, CwlA, which belongs to the NlpC/P60 family of endopeptidases and hydrolyses cross-linked PG between daughter cells to allow cell separation. We identified CwlA as the first PrkC substrate in C. difficile. We demonstrated that PrkC-dependent phosphorylation inhibits CwlA export, thereby controlling hydrolytic activity in the cell wall. High levels of CwlA at the cell surface led to cell elongation, whereas low levels caused cell separation defects. Thus, we provided evidence that the STK signaling pathway regulates PGH homeostasis to precisely control PG hydrolysis during cell division.

## INTRODUCTION

**FIG 1:**
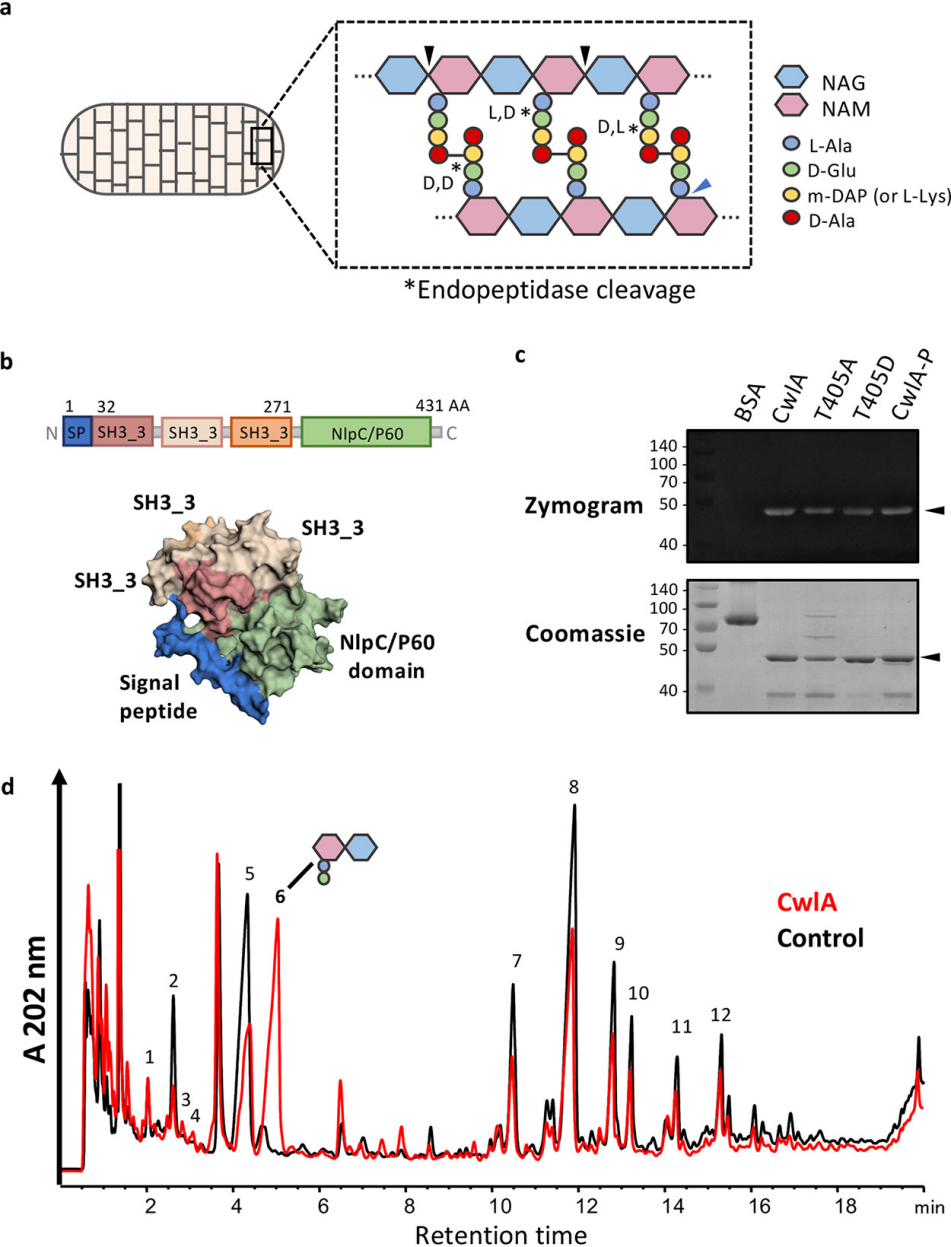
CwlA is γ-d-Glu-mDAP-endopeptidase. (a) Schematic representation of peptidoglycan sacculus, a polymer of β-(1,4)-linked *N-*acetylglucosamine (NAG) (blue) and *N*-acetylmuramic acid (NAM) glycan strands (pink). Enzymes that hydrolyze peptidoglycan are classified as glycosidases (black arrow), amidases (blue arrow), and endopeptidases (*) depending on where they cleave. (b) Structural prediction of CwlA colored by domains. Signal peptide is in blue (1 to 31 amino acids), three SH3_3 domains (also named SH3b; pfam08239) are in pink (32 to 95), light pink (114 to 178), and orange (207 to 271), and the catalytic domain NlpC/P60 (pfam00877) is in green. Structural prediction was generated with Phyre 2.0 based on the alignment with c6biqA, c3npfB, and c3h41A. Highlighted domains were determined with EzMol 2.1. (c) Detection by zymogram of hydrolytic activities of purified proteins: BSA (negative control), CwlA, CwlA-T405A (T405A), CwlA-T405D (T405D), and PrkC-dependent phosphorylation of CwlA (CwlA-P). The gel contained 1 mg/ml purified PG as a substrate. Arrows indicate the positions of the hydrolytic bands. (d) RP-HPLC separation of PG fragments released from C. difficile PG after incubation with mutanolysin (control, black) or His_6_-CwlA (red).

Peptidoglycan (PG), the major component of the bacterial cell wall (CW), protects bacteria from environmental stresses and contributes to virulence and antibiotic resistance (1). PG is classically made of glycan chains of alternating *N-*acetylglucosamine (NAG) and *N*-acetylmuramic acid (NAM) cross-linked by short stem peptides attached to NAM (2). PG remodeling requires three types of hydrolases (PGH, or autolysins): (i) glycosidases hydrolyze the glycosidic linkages, (ii) amidases cleave the amide bond between NAM and l-alanine residue, and (iii) endopeptidases cleave amide bonds between amino acids within the PG peptidic chains (3) (Fig. 1a). PGHs are involved in fundamental aspects of bacterial physiology, such as CW expansion, turnover, and recycling during growth, cell separation, and autolysis (4–6). Different strategies are employed by bacteria to coordinate PG synthesis and degradation. Typical regulation occurs through a signal, such as iron concentration, that regulates the transcription of the autolysin-encoding gene *isdP* in Staphylococcus lugdunensis (7) or through two-component systems, such as WalK-WalR, that monitor the expression of the *lytE* and *cwlO* genes encoding endopeptidases in Bacillus subtilis by sensing and responding to their cleavage products (8). PGH activity can also be directly regulated, for instance, through the control of their cell surface localization. The FtsEX complex, associated with the divisome, recruits and activates Streptococcus pneumoniae PcsB (9–11) and B. subtilis CwlO (12) hydrolases involved in cell division and elongation. In Escherichia coli, the interaction of FtsEX with EnvC activates cell separation amidases (12, 13). Alternatively, proteolysis modulates E. coli Meps (14) and Mycobacterium tuberculosis RipA (15) hydrolase activities. Post-translational modifications also provide an efficient and fine-tunable way to regulate PGH activity. The tyrosine kinase CspD phosphorylates LytA, a pneumococcal autolysin enhancing its amidase activity (16), whereas *O*-glycosylation of the *Lactobacillus plantarum N*-acetylglucosaminidase decreases its activity (17). The synthesis or activity of several key enzymes of PG metabolism is also regulated by Hanks-type serine/threonine kinases (STKs). In all firmicutes, a *trans*-membrane kinase with an extracellular domain containing penicillin-binding and STK-associated (PASTA) repeats is present. PASTA domains interact with β-lactam antibiotics (18), non-cross-linked PG fragments (muropeptides) (19), and lipid II (20, 21). Inactivation of genes encoding PASTA-STKs is associated with cell division defects, changes in CW metabolism, and modified susceptibility to CW-targeting antibiotics (22–24). In S. pneumoniae, STK allows localization of the LytB PGH at the septum through their PASTA repeats (25). However, only a few STK substrates have been identified so far.

Clostridioides difficile contains two STKs of the Hanks family, the PASTA kinase, PrkC, and a second kinase, CD2148, and one PP2C-type phosphatase (STP), which dephosphorylates the Hanks-kinase substrates. C. difficile is a Gram-positive, spore-forming, anaerobic bacterium and is the leading cause of antibiotic-associated nosocomial diarrhea (26). The incidence and severity of C. difficile infection have recently increased, representing a challenging threat to human health (27, 28). We previously phenotypically characterized the C. difficile Δ*prkC* mutant and highlighted the role of PrkC in CW homeostasis. A Δ*prkC* mutant exhibited modifications in cell morphology and septum formation and was also more sensitive to antimicrobial compounds that target the CW (29). In this work, we identified and characterized a PGH, CD1135 (renamed CwlA; see below). We showed that CwlA functions as an endopeptidase and hydrolyses PG between daughter cells to allow cell separation. We found that CwlA is phosphorylated by PrkC and demonstrated that PrkC-dependent phosphorylation controls the export of CwlA required for cytokinesis. This represents a novel and original mechanism for CW hydrolysis regulation by STK phosphorylation.

## RESULTS

### CD1135 (CwlA) is a γ-d-Glu-mDAP-endopeptidase.

CD1135 contains a signal sequence, three putative SH3_3 (also named SH3b) domains, which are predicted to contribute to PG recognition and binding (30–32), and a catalytic domain of the NlpC/P60 family (Fig. 1b). Proteins containing NlpC/P60 domains are ubiquitous papain-like cysteine peptidases hydrolyzing the NAM-l-alanine or d-γ-glutamyl-*meso*-diaminopimelate (DAP) linkages (33). Three conserved residues are involved in catalysis: Cys, His, and a polar residue (His, Asp, or Gln). The alignment of the NlpC/P60 domain of CD1135 with the endopeptidases LytF, LytE, CwlS, and YkfC from B. subtilis and YkfC from B. cereus revealed that these residues are fully conserved (see Fig. S1 in the supplemental material). To provide evidence that CD1135 functions as a PGH, the hydrolytic activity of His_6_-tagged CD1135 protein was examined by zymogram with purified C. difficile PG. A clear lytic band with a molecular weight around 46 kDa, corresponding to His_6_-CD1135, was detected (Fig. 1c), confirming the PG-degrading activity of CD1135. To characterize more precisely this activity and the specificity of the CD1135 enzyme, we incubated the purified protein with PG of the C. difficile 630Δ*erm* strain. After incubation, no soluble PG fragment was detected by reverse-phase high-pressure liquid chromatography (RP-HPLC) analysis (data not shown), and the insoluble PG fraction was further digested with mutanolysin to reveal potential cleavages inside the cross-linked PG. The resulting muropeptide profile obtained by RP-HPLC was clearly distinct from the control muropeptide profile of PG digested with mutanolysin alone (Fig. 1d). In particular, PG incubated with CD1135 and mutanolysin contained a major muropeptide (peak 6) present only in small amounts in the PG digested with mutanolysin alone. Mass spectrometry (MS) analysis showed that peak 6 contained deacetylated disaccharide-dipeptide (Table 1), revealing cleavage by CD1135 of the chemical bond between γ-d-Glu and mDAP inside the PG stem peptides. Concomitantly, a decrease of several muropeptides was observed in PG incubated with CD1135 compared to the control (Fig. 1d), including monomers identified as deacetylated disaccharide-tetrapeptides with d-Ala (peak 5) or Gly (peak 2) in position 4 of the stem peptide by MS analysis (Table 1). All these results indicate that CD1135 can hydrolyze stem peptides inside the cross-linked PG and that CD1135, renamed CwlA, is a γ-d-Glu-mDAP-endopeptidase.

**TABLE 1:** Proposed structures deduced from *m/z* values of muropeptides resulting from the digestion of C. difficile PG by purified His_6_-CD1135 (CwlA) followed by mutanolysin

Peak^*a*^	Muropeptide^*b*^	*m/z*		Charge (*z*)
		Expected	Measured	
1	Tri (deAc)	415.1878	415.1876	2
2	Tri-Gly (deAc)	443.6985	443.6977	2
3	Tri-Ala-mDAP (deAc)	536.7487	536.7463	2
4	Di	699.2936	699.2934	1
5	Tetra (deAc)	450.7064	450.7062	2
6	Di (deAc)	657.2831	657.2833	1
7	Tri-Tri-Gly (deAc X2)	848.8733	848.8749	2
8	Tri-Tetra (deAc X2)	855.8811	855.8809	2
9	Tri-Tetra (deAc X2)	855.8811	855.8823	2
10	Tetra-Tetra (deAc X2)	891.3997	891.4020	2
11a	Tetra-Tri-Tri (deAc X3)	841.0398	841.0406	3
11b	Tetra-Tetra (deAc X2)	891.3997	891.4010	2
12	Tri-Tetra-Tetra (deAc X3)	864.7189	864.7201	3

a Peak numbers correspond to those in Fig. 1d.

b Di, disaccharide dipeptide (l-Ala-d-Glu); Tri, disaccharide tripeptide (l-Ala-d-iGlu-mDAP); Tetra, disaccharide tetrapeptide (l-Ala-d-iGlu-mDAP-d-Ala); disaccharide, GlcNAc-MurNAc; deAc, deacetylation on GlcNAc, iGlu, isoglutamic acid; mDAP, mesodiaminopimelic acid, Gly, glycine.

10.1128/mBio.00519-21.1 FIG S1 CD1135 (CwlA) belongs to the NlpC/P60 family. (a) Alignment of CD1135 with conserved regions of NlpC/P60 domains of B. cereus YkfC (BcYkfC), B. subtilis YkfC (BsYkfC), LytF (BsLytF), LytE (BsLytE), and CwlS (BsCwlS) contain the essential residues for catalysis (Cys, His, Asp; black). Conserved residues are in red boxes and similar residues in red characters. (b) Schematic representation of B. subtilis LytF, LytE, CwlS, and YkfC B. cereus YkfC and CD1135. Download FIG S1, PDF file, 0.6 MB. Copyright © 2021 Garcia-Garcia et al. 2021 Garcia-Garcia et al. https://creativecommons.org/licenses/by/4.0/ This content is distributed under the terms of the Creative Commons Attribution 4.0 International license.

### Changes in CwlA abundance lead to cell separation defects during growth.

**FIG 2:**
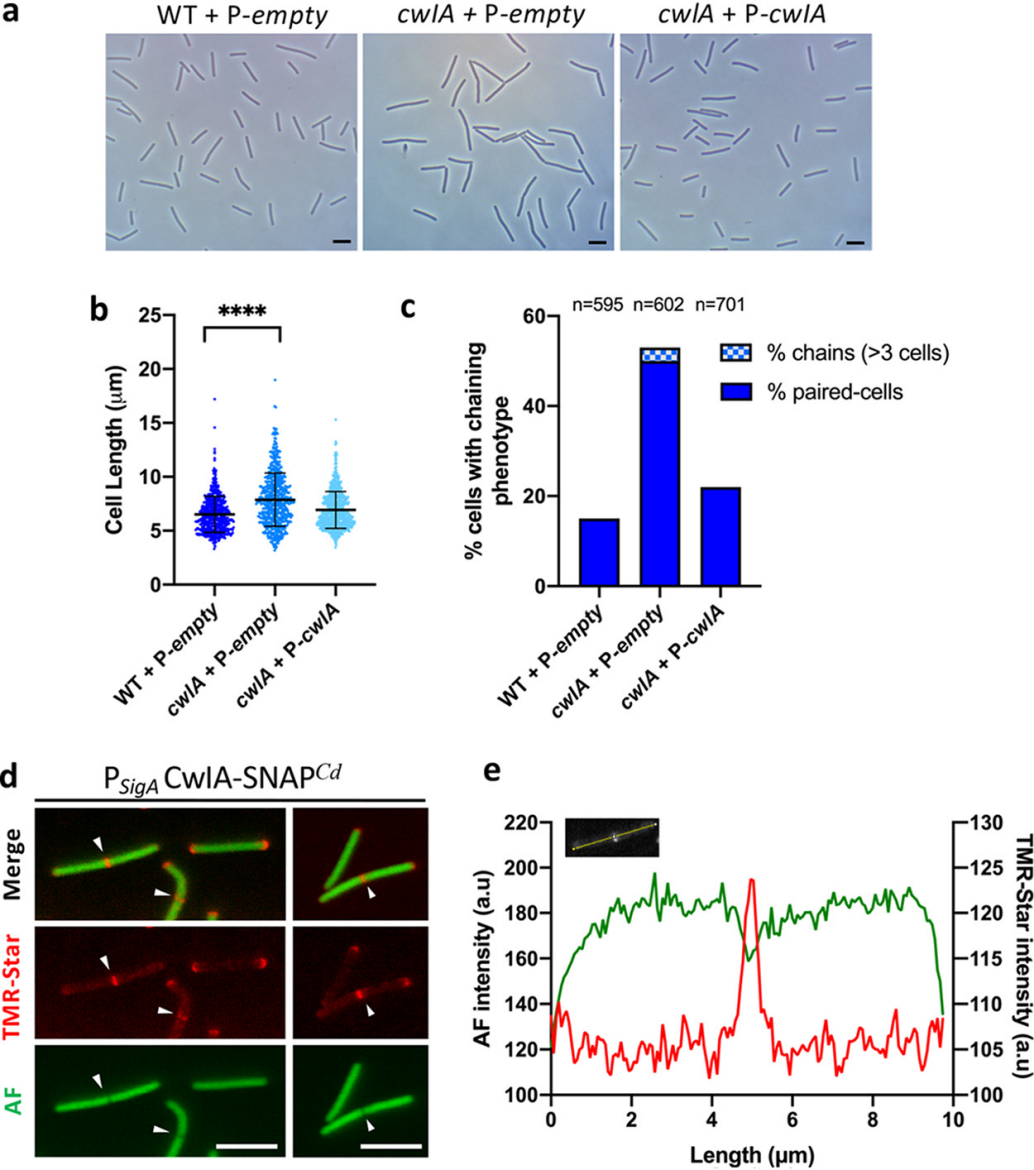
CwlA is involved in cell division. (a) Phase-contrast images of exponentially growing C. difficile cells of 630Δ*erm* (WT) + P*-empty*, *cwlA* + P*-empty*, and *cwlA* + P-*cwlA* strains. Scale bar, 5 μm. (b) Scatterplots showing cell length, with the median and standard deviation (SD) from each distribution indicated by a black line. *P* value was determined by two-sided Mann-Whitney *U* tests (****, *P < *0.0001); 595 (WT + P*-empty*), 602 (*cwlA* + P*-empty*), and 701 (*cwlA* + P*-cwlA*) cells were counted. (c) Percentage of cells harboring a chaining phenotype. The *n* value represents the number of cells analyzed in a single representative experiment. The images are representative of experiments performed in triplicate. (d) CwlA-SNAP*^Cd^* protein fusion and its localization. A plasmid carrying the CwlA-SNAP*^Cd^* fusion under the control of its own promoter was introduced into the *cwlA* strain. Merge images (upper), TMR-Star fluorescent signal (middle), and auto-fluorescence AF (lower) are shown. Scale bar, 5 μm. (e) Quantification of fluorescence in arbitrary units (a.u) along the cell for a typical cell. AF, green; TMR-Star, red.

To determine the role of this PGH in C. difficile, a *cwlA* mutant was generated. This mutant grew similarly to the 630Δ*erm* wild-type (WT) strain (Fig. S2a). Using phase-contrast microscopy, we revealed, during exponential growth, a slight but significant increase in cell length for the mutant (7.8 ± 2.4 μm) compared to the WT (6.5 ± 1.6 μm) cells (Fig. 2a and b). Fifty percent of the mutant cells also existed as unseparated paired cells compared with only 15% for the WT strain (Fig. 2a and c). Cell length and cell separation were almost completely restored in a *cwlA* mutant complemented with a plasmid harboring the *cwlA* gene expressed from its own promoter (Fig. 2a to c). This result strongly suggests that CwlA is required to cleave PG at the septum and allow separation of daughter cells. To determine the localization of CwlA, we constructed a plasmid encoding a CwlA-SNAP*^Cd^* fusion produced under the control of its own promoter. The CwlA-SNAP*^Cd^* fusion protein localized mainly at the septum of dividing cells (Fig. 2d) but also at the poles of nondividing cells. However, transmission electron microscopy (TEM) analysis of the WT, *cwlA* mutant, and complemented cells revealed no difference in septum and CW thickness (Fig. S2b). These results indicate that the PGH CwlA present at the septum is involved in cytokinesis.

10.1128/mBio.00519-21.2 FIG S2 Growth and TEM of *cwlA* mutant. (a) Growth curves of *cwlA* mutant compared to WT strain in TY. (b) Transmission electron microscopy showing septum thickness and cell wall width of 630Δ*erm* + P-*empty* (WT), *cwlA* + P-*empty*, and *cwlA* + P-*cwlA* strains. Middle and right columns, higher magnifications (100 nm) of the division septa (highlighted by a black square) and cell wall, respectively. Download FIG S2, PDF file, 0.6 MB. Copyright © 2021 Garcia-Garcia et al. 2021 Garcia-Garcia et al. https://creativecommons.org/licenses/by/4.0/ This content is distributed under the terms of the Creative Commons Attribution 4.0 International license.

**FIG 3:**
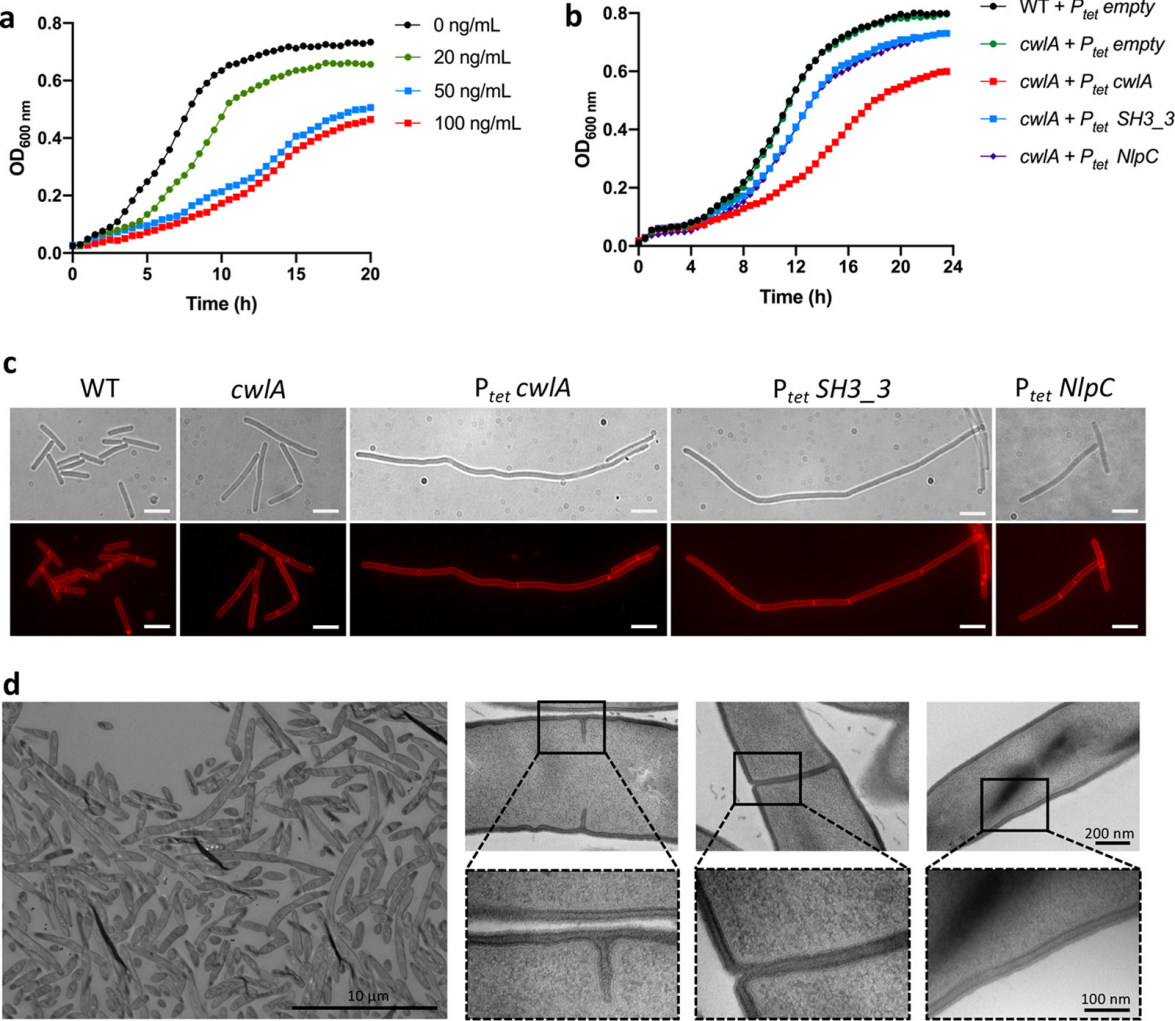
Overexpression of *cwlA* inhibits growth and produces filamented cells. (a) Growth curves of *cwlA* mutant carrying pDIA6103-P*_tet_ cwlA* in TY at 0, 20, 50, and 100 ng/ml ATc. (b) Growth curves showing the effect of overexpression of *cwlA* and *cwlA* overexpressing only the SH3_3 domains (*cwlA* + P*_tet_ SH3_3*) or the catalytic domain NlpC/P60 (*cwlA* + P*_tet_ NlpC*) when 50 ng/ml ATc was added at the beginning of growth. (c) Phase-contrast images (upper) and fluorescence microscopy (lower) of cells overexpressing full-length *cwlA* or the NlpC/P60 and SH3_3 domains stained with FM4-64. Scale bars, 5 μm. (d) Transmission electron micrographs of septal regions and cell wall from cells overexpressing *cwlA*. Lower panels show higher magnifications (100 nm) of the division septa or cell wall, highlighted by a black square. Growth curves and images are representative of experiments performed in triplicate.

We then expressed *cwlA* under the control of the P*_tet_* inducible promoter on a plasmid. Growth of the *cwlA* mutant containing pDIA6103-P*_tet_ cwlA* was impaired in the presence of ATc (Fig. 3a and b). In addition, we observed a filamentation phenotype upon induction of *cwlA* expression with 50 ng/ml ATc (Fig. 3c and Fig. S3). In contrast, neither WT nor *cwlA* mutant strains harboring an empty plasmid were similarly affected in morphology or growth (Fig. 3b and c and Fig. S3). A filamentation phenotype in strains that overexpress a gene encoding an endopeptidase is usually not observed (34). Staining with FM4-64 and TEM revealed few septa in these filamented cells, suggesting that division was impaired prior to constriction (Fig. 3c and d). We then compared the PG structure of the WT strain, the *cwlA* mutant, and cells overexpressing *cwlA*. Purified PG was digested with mutanolysin to generate muropeptides for RP-HPLC analysis. However, no significant differences were detected between these strains, suggesting that the phenotype observed in cells overexpressing *cwlA* is not the result of higher CwlA activity (Fig. S4). Therefore, the observed filamentation might be due to a saturation of the SH3_3 binding sites by CwlA, leading to an impaired anchoring to the surface of other PGH-containing SH3_3 domains. To test this possibility, we expressed, under the control of the P*_tet_* promoter, the 5′ part of the *cwlA* gene corresponding to the three SH3_3 domains associated with the signal peptide. The *cwlA* mutant overexpressing this truncated gene also exhibited an elongation phenotype but a growth defect reduced compared to that of the overexpression of the complete *cwlA* gene (Fig. 3b and c and Fig. S3). To exclude a possible role of the NlpC/P60 domain in cell elongation, we also expressed, under the control of the P*_tet_* promoter, the 3′ part of the *cwlA* gene corresponding to this catalytic domain associated with the signal peptide. While this *cwlA* P*_tet-_*NlpC strain grew similarly to the strain carrying only the three SH3_3 domains, the phenotype of cell elongation was almost abolished (Fig. 3b and c and Fig. S3). Thus, saturation of the SH3_3 binding sites is likely responsible for the cell division defect observed upon *cwlA* overexpression.

10.1128/mBio.00519-21.3 FIG S3 Morphology of cells overexpressing *cwlA*. (a) Phase-contrast images of C. difficile cells of WT + P*_tet_ empty*, *cwlA* + P*_tet_ empty*, *cwlA* + P*_tet_ cwlA*, *cwlA* + P*_tet_ SH3_3*, and *cwlA* + P*_tet_ NlpC* strains. Scale bar, 5 μm. (b) Scatter plots showing the distribution of cell length. Two-sided Mann–Whitney *U* tests (****, *P < *0.0001; *, *P < *0.05) for 148 (WT + P*_tet_ empty*), 223 (*cwlA* + P*_tet_ empty*), 219 (*cwlA* + P*_tet_ cwlA*), 208 (*cwlA* + P*_tet_ SH3_3*), and 180 (*cwlA* + P*_tet_ NlpC*) cells counted in a single representative experiment. Experiments were performed in triplicate. Download FIG S3, PDF file, 2.1 MB. Copyright © 2021 Garcia-Garcia et al. 2021 Garcia-Garcia et al. https://creativecommons.org/licenses/by/4.0/ This content is distributed under the terms of the Creative Commons Attribution 4.0 International license.

10.1128/mBio.00519-21.4 FIG S4 Profiles of muropeptides of *cwlA* strains. (a) Schematic of peptidoglycan (PG) isolation and digestion for RP-HPLC analysis and mutanolysin treatment of cross-linked PG generate muropeptides species. (b) RP-HPLC separation profile of muropeptides from WT (P*_tet_ empty*), *cwlA* (P*_tet_ empty*), *cwlA* + P*_tet_ cwlA*, and *cwlA* + P*-cwlA* (*cwlA* + pMTL84121*-cwlA*) strains. The profiles were superimposed and the peaks were numbered according to Cuenot et al. (29). Peak numbers 1, 4, 6, and 7 correspond to monomers, and peak numbers 11, 13, 14, 15, 17, 18, 19, and 21 correspond to dimers. The abundance of each molecule is correlated with the area present under the corresponding peak. Download FIG S4, PDF file, 0.5 MB. Copyright © 2021 Garcia-Garcia et al. 2021 Garcia-Garcia et al. https://creativecommons.org/licenses/by/4.0/ This content is distributed under the terms of the Creative Commons Attribution 4.0 International license.

### *CD2148* and *prkC* mutants show similar phenotypes for lack and excess of CwlA, respectively.

**FIG 4:**
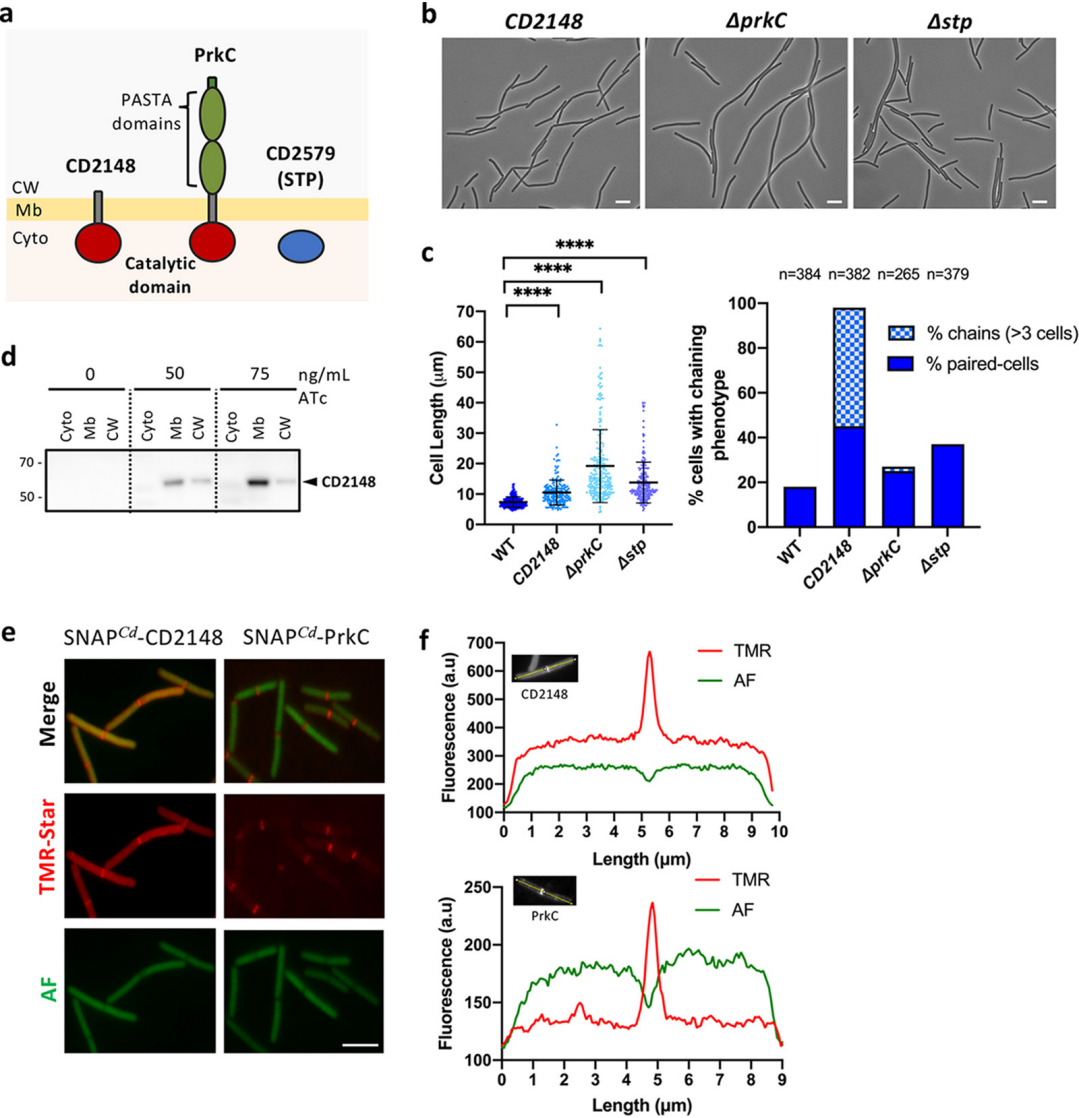
*CD2148* and Δ*prkC* mutants show phenotypes similar to lack or overproduction of CwlA. (a) Schematic representation of C. difficile STKs and STP. (b) Phase-contrast images of *CD2148*, Δ*prkC*, and Δ*stp* mutants. Scale bar, 5 μm. (c) Scatterplots showing cell length of C. difficile STK mutants (left) and percentage of cells harboring a chaining phenotype (right). *P* values were determined by two-sided Mann-Whitney *U* tests (****, *P < *0.0001); 320 (WT), 188 (*CD2148*), 251 (Δ*prkC*), and 231 (Δ*stp*) cells were counted. (d) Western blot showing the location of SNAP*^Cd^*-CD2148 in the different cell fractions: cytoplasm (Cyto), membrane (Mb), and cell wall (CW). (e) Localization of SNAP*^Cd^*-CD2148 and SNAP*^Cd^*-PrkC protein fusions. Merge images (upper), TMR-Star fluorescent signal (middle), and autofluorescence AF (lower) are shown. Scale bar, 5 μm. (f) Quantification of fluorescence in arbitrary units (a.u) along the major axis for a typical cell. The images are representative of experiments performed in triplicate.

The C. difficile genome contains genes encoding two STKs, the PASTA-kinase PrkC and a second kinase, CD2148, and the associated PP2C-type phosphatase (STP) (Fig. 4a and Fig. S5a). We previously obtained a *prkC* deletion mutant (29). Here, we constructed an *stp* deletion as well as a *CD2148* mutant in both 630Δ*erm* and Δ*prkC* strains. Strains complemented for *CD2148* expressed from its own promoter and *stp* expressed under the control of a P*tet* promoter (Fig. S5a and b) were also obtained. Since *prkC* and *stp* form an operon (29), we also verified by quantitative reverse transcription-PCR (qRT-PCR) that the inactivation of *stp* or *prkC* did not modify the expression of the other adjacent gene to exclude the existence of a polar effect (Fig. S5c). Interestingly, *prkC* inactivation led to cell filamentation (29) (Fig. 4b and c), a phenotype reminiscent of bacteria overexpressing *cwlA* (Fig. 3c). In contrast, a *CD2148* mutant exhibited a slight increase in cell length (10.5 ± 4.0 μm) compared to the WT strain, similar to the *cwlA* mutant, and a cell separation defect more accentuated than that in the *cwlA* mutant (Fig. 4b and c and Fig. S5d and e). In the *CD2148* mutant, 98% of the cells presented a chaining phenotype, with 53% of cells found as chains of more than 3 unseparated cells (Fig. 4c, right). The WT phenotype was recovered upon introduction of a plasmid containing the *CD2148* gene under the control of its own promoter (Fig. S5d and e). The *stp* deletion mutant showed a filamentation phenotype with an average size corresponding to 13.7 ± 6.6 μm, and 37% of cells were found as paired cells (Fig. 4b and c). The WT phenotype was restored in an *stp* mutant containing a pDIA6103-P*_tet_*-*stp* plasmid after induction of *stp* expression in the presence of 15 ng/ml of ATc (Fig. S5d and e).

10.1128/mBio.00519-21.5 FIG S5 Functional characterization of CD2148 and STP. (a) Genetic organization of the *CD2148* locus. TSS mapping experiment indicated the presence of a σ^A^-dependent promoter upstream of *CD2148* and *CD2149* (72). The −35 and −10 boxes as well as the TSS are indicated in boldface. (b) Growth curves of *CD2148* mutant compared to WT strain and the complemented strain *CD2148 *+* *P-*CD2148* in TY. (c) qRT-PCR analysis of *stp* and *prkC* expression in Δ*stp* and Δ*prkC* mutants compared to the WT strain. The result presented is the mean of the data obtained with 4 independent RNA samples. (d) Phase-contrast images of WT (630Δ*erm* + pMTL84121), *CD2148 (CD2148*::*erm +* pMTL84121), *CD2148 *+* *P-*CD2148* (*CD2148*::*erm +* pMTL84121-*CD2148*), Δ*stp* (Δ*stp +* pDIA6103), and Δ*stp +* P*-stp* (Δ*stp +* pDIA6103-*stp*) cells in TY at exponential phase. (e) Scatter plots showing cell length (left) and percentage of cells harboring a chaining phenotype (right). *P* values were determined by two-sided Mann–Whitney *U* tests (****, *P < *0.0001) for 575 (WT + P-*empty*), 647 (*CD2148 + *P-*empty*), 584 (*CD2148 *+* *P-*CD2148*), 525 (Δ*stp +* P-*empty*), and 415 (Δ*stp +* P-*stp*) cells counted. (f, upper) Transmission electron microscopy showing division septa (red arrows). Scale bars, 2 μm. (Lower) Higher magnifications (200 nm) of septum thickness. Download FIG S5, PDF file, 1.6 MB. Copyright © 2021 Garcia-Garcia et al. 2021 Garcia-Garcia et al. https://creativecommons.org/licenses/by/4.0/ This content is distributed under the terms of the Creative Commons Attribution 4.0 International license.

In contrast to PrkC, the CD2148 kinase does not contain an extracellular PASTA domain. However, the presence of a transmembrane segment at the C terminus of the protein suggests an association with the membrane (Fig. 4a) (29, 35, 36). To analyze the cellular localization of CD2148, we performed a cell fractionation to separate cytoplasmic, membrane, and CW fractions using a strain carrying a plasmid with a P*_tet_* SNAP*^Cd^*-*CD2148* fusion. Western blots using antibodies raised against SNAP revealed that CD2148 is mainly found in the membrane (Fig. 4d). Localization of CD2148 was also determined using the P*_tet_* SNAP*^Cd^*-*CD2148* fusion. After induction, we detected the SNAP-CD2148 fusion protein in the cytoplasm with an enrichment at mid-cell (Fig. 4e and f). This localization pattern is slightly different from that of PrkC and CwlA, which mostly localized at the cell septum (Fig. 2d and 4e and f). These results indicated that CD2148, similar to CwlA, partially localizes at the cell septum, in agreement with its role in cell separation.

### CwlA is phosphorylated *in vivo* and *in vitro* by PrkC.

**FIG 5:**
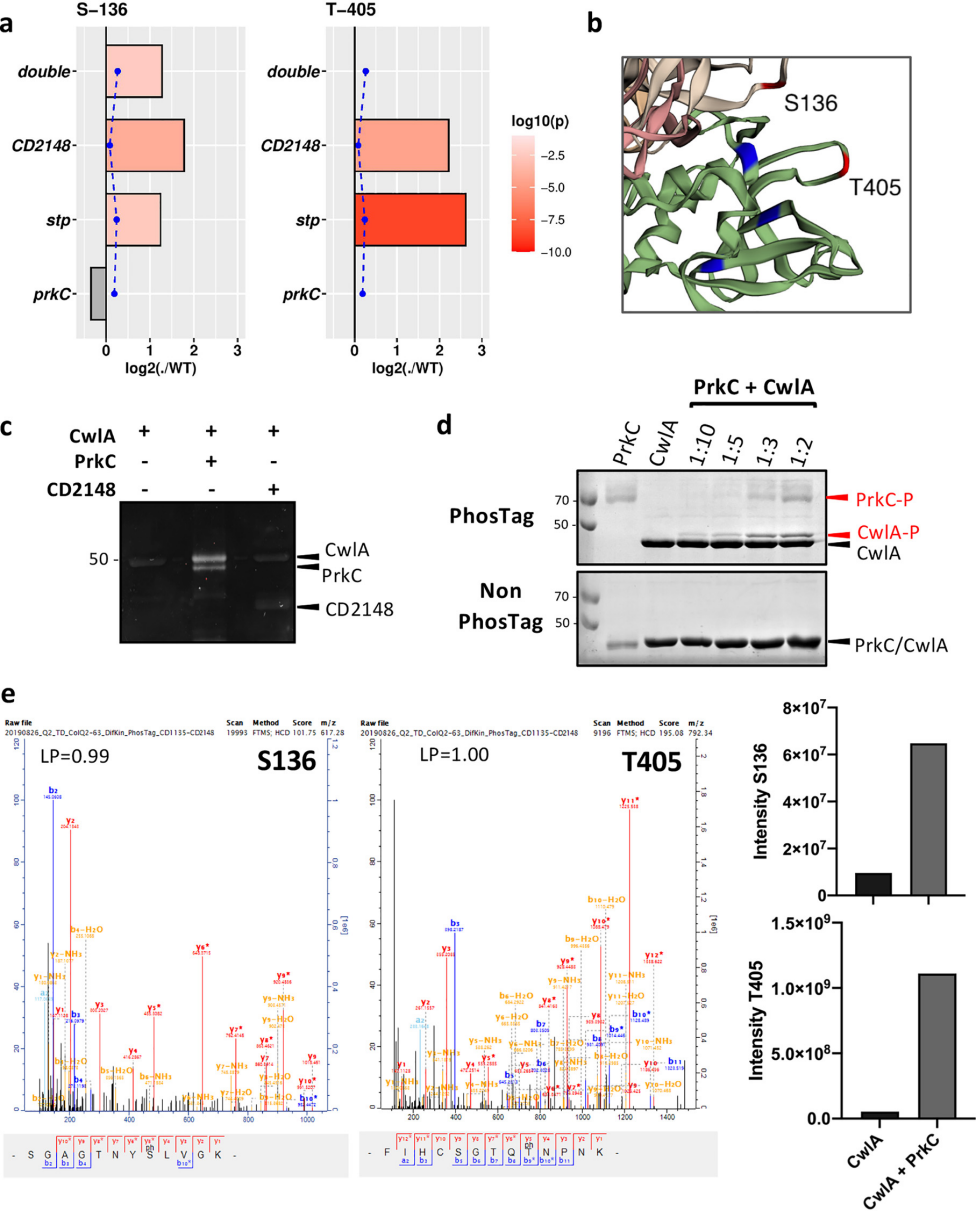
CwlA is phosphorylated by PrkC. (a) *In vivo* phosphorylation of CwlA as detected in phosphosproteomic data. Bar graphs represent fold change of the phosphosite serine 136 (S136) or threonine 405 (T405) among Δ*prkC*, *Δstp*, *CD2148*, and Δ*prkC CD2148* (double) mutants compared to the WT. The absence of bars means that no phosphopeptides were detected. Significance is represented in red by log_10_ (*P* value), and not significant (ns) is represented in gray. The blue dotted line represents fold change of peptide amounts. (b) Phosphorylated residues S136 and T405 (in red) are in close proximity to the three conserved catalytic residues: Cys, His, and Asp (in blue). (c) *In vitro* phosphorylation assay of CwlA by the purified kinase domains of STKs of C. difficile, PrkC, and CD2148. Phosphorylation was visualized by Phos-Tag fluorescent gel stain reagents. (d) *In vitro* phosphorylation assay of CwlA by PrkC at different ratios of kinase to substrate. Phosphorylation was visualized by Phos-Tag acrylamide for the separation of phosphorylated and nonphosphorylated proteins. (e) Mass spectrometry detection of the phosphorylation sites on CwlA after *in vitro* phosphorylation reactions performed without and with the kinase domain of PrkC. LP is the localization probability of the identified phosphosite.

Since PrkC and CD2148 are STKs, we compared the level of phosphorylation of CwlA in the WT, Δ*prkC*, *CD2148*, Δ*prkC CD2148*, and Δ*stp* strains. CwlA was phosphorylated at S136 in all strains. However, a higher intensity of S136 phosphorylation was detected in the Δ*stp*, *CD2148*, and Δ*prkC CD2148* double mutant, while S136 was slightly less phosphorylated in the Δ*prkC* mutant (Fig. 5a, left). A second residue, T405, was also found phosphorylated in the WT strain as well as in the Δ*stp* and *CD2148* mutants, while its phosphorylation was abolished in the Δ*prkC* mutant and in the Δ*prkC CD2148* double mutant (Fig. 5a, right). These results indicated that T405 phosphorylation is PrkC dependent *in vivo.* Furthermore, we observed that the level of phosphorylation of T405 was significantly higher (more than 4-fold) in the Δ*stp* and *CD2148* mutants than the WT strain, while the peptide amounts were similar. STP is probably involved in the dephosphorylation of CwlA. In addition, the increased phosphorylation level of T405 detected in the *CD2148* mutant disappeared in the *CD2148* Δ*prkC* double mutant. This result indicated that the effect of *CD2148* inactivation on T405 phosphorylation was mediated through PrkC and that the role of the second STK, CD2148, was more complex than expected (see below). Moreover, additional phosphorylated residues were detected in the *CD2148* and Δ*stp* mutants (Fig. S6a). This may contribute to the hyperphosphorylation of CwlA in these strains.

10.1128/mBio.00519-21.6 FIG S6 *In vivo* and *in vitro* phosphorylation of CwlA. (a) Surface representation of CwlA showing phosphorylated residues identified *in vivo* in the STKs/STP mutants with a good localization probability (LP > 0.75). Phosphosites S-136 and T-405 are highlighted in black and green, respectively. CwlA is colored by domains (blue, signal peptide; pink, SH3_3.1; light pink, SH3_3.2; orange, SH3_3.3; and green, NlpC/P60), and phosphosites are labeled in red. (b) *In vitro* phosphorylation assay of CwlA, CwlA-T405A, and CwlA-T405D by PrkC or CD2148 and visualized by Phos-Tag acrylamide. The red arrow indicates phosphorylated CwlA. Download FIG S6, PDF file, 1.5 MB. Copyright © 2021 Garcia-Garcia et al. 2021 Garcia-Garcia et al. https://creativecommons.org/licenses/by/4.0/ This content is distributed under the terms of the Creative Commons Attribution 4.0 International license.

To confirm the specificity of CwlA phosphorylation *in vitro*, we purified the catalytic domain of PrkC and CD2148. Purified CwlA was incubated with the purified kinase domain (KD) of PrkC or CD2148 at a 1:10 ratio in the presence of cold ATP. To determine the phosphorylation status of CwlA (Fig. 5c), a Phos-Tag fluorescent gel stain method allowing the specific detection of phosphorylated proteins was used (37). When CwlA was incubated with PrkC, two bands with similar molecular weights were detected. The upper band corresponded to CwlA (46.1 kDa) and the lower band to the kinase domain of PrkC (45 kDa), which is known to be autophosphorylated (36, 38, 39). In contrast, no fluorescence was detected when CwlA was incubated alone or with CD2148 (Fig. 5c). To detect phosphorylated isoforms of purified His_6_-CwlA, we further used Phos-Tag acrylamide gel to separate phosphorylated and nonphosphorylated forms (40). PrkC was the only kinase that efficiently phosphorylated CwlA *in vitro* (Fig. 5d and Fig. S6b). The phosphorylation of S136 and T405 residues detected *in vivo* was then confirmed *in vitro* by mass spectrometry (Fig. 5e). However, the level of phosphorylation of Ser136 by PrkC *in vitro* was 100-fold less than the level of phosphorylation of T405 (Fig. 5e), in agreement with the results obtained *in vivo* (Fig. 5a). Indeed, while Ser136 was phosphorylated *in vivo* in all strains, T405, located within the NlpC/P60 domain, was more efficiently phosphorylated *in vitro*, and its phosphorylation *in vivo* was strictly PrkC dependent. To confirm whether T405 was specifically phosphorylated by PrkC, this residue was replaced with the nonphosphorylatable residue alanine (T405A) or the phospho-mimetic residue aspartate (T405D) by site-directed mutagenesis. The CwlA-T405A and CwlA-T405D proteins were then purified and tested *in vitro* for PrkC-mediated phosphorylation. In both cases, the phosphorylation of CwlA-T405A and CwlA-T405D by PrkC *in vitro* was abolished, as determined by Phos-Tag acrylamide (Fig. S6b). Altogether, these results indicated that T405 is the major site of STK-dependent phosphorylation of CwlA and that this phosphorylation is specific for PrkC.

### Phosphorylation at T405 does not impact CwlA PGH activity.

Based on the three-dimensional (3D) structure prediction of CwlA obtained with Phyre 2.0 (41), the phosphorylated residues S136 and T405 are likely located in the second SH3_3 and in the NlpC/P60 domain, respectively (Fig. S6a). T405 is in the vicinity of the three conserved catalytic residues, suggesting that PrkC-dependent phosphorylation regulates CwlA activity (Fig. 5b and Fig. S1b). To determine if the phosphorylation could modify the PG-degrading activity, the purified CwlA-T405A and CwlA-T405D proteins or the CwlA protein phosphorylated *in vitro* by PrkC were tested by zymogram. Neither the substitution of T405 nor the phosphorylation of CwlA by PrkC affected the hydrolytic activity of these proteins in zymograms (Fig. 1c). These results indicate that T405 phosphorylation is neither beneficial nor detrimental for the activity of CwlA.

### Effect of T405 phosphomimetic and nonphosphorylatable mutations of CwlA *in vivo*.

**FIG 6:**
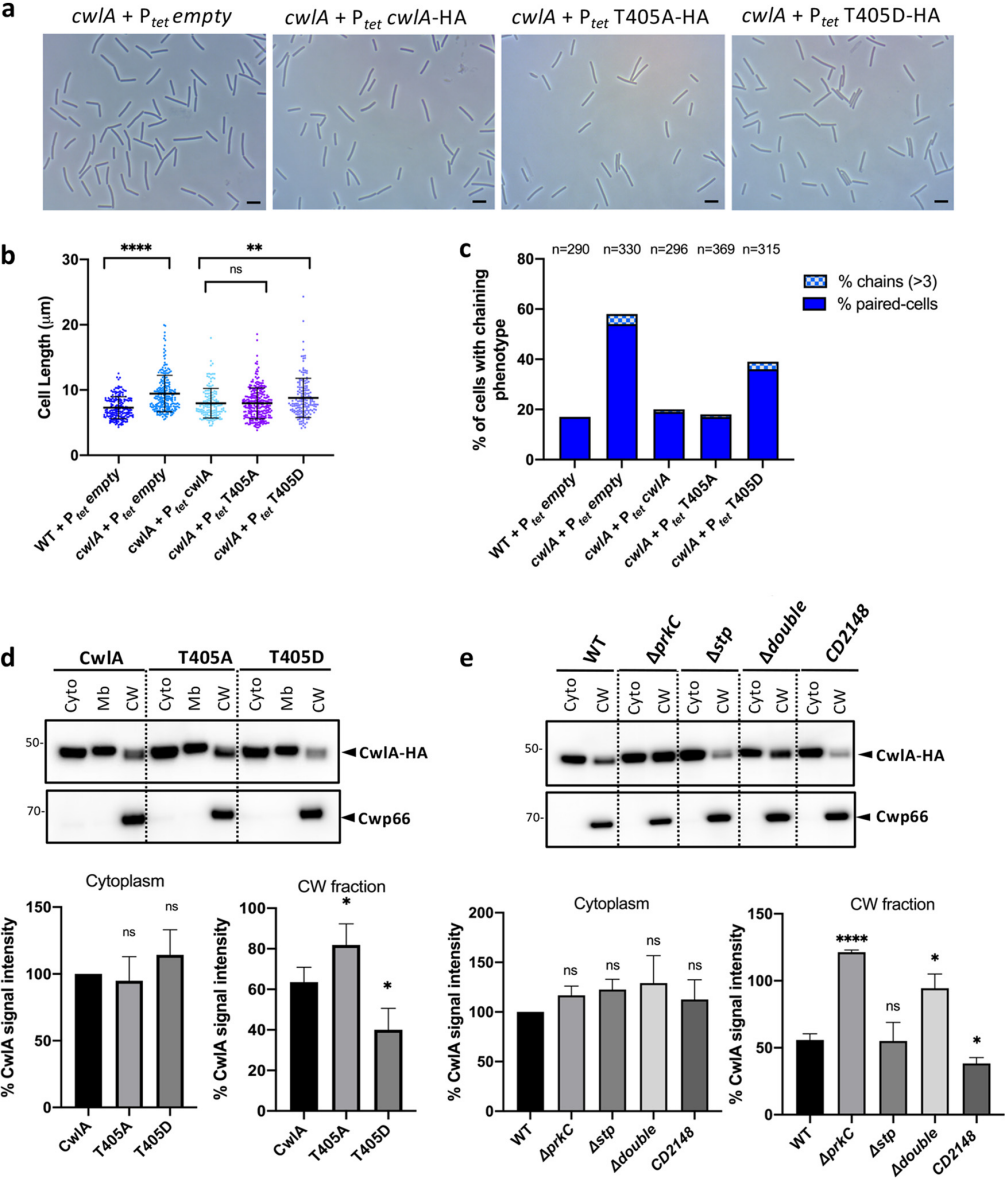
Effect of CwlA phosphorylation on its cell wall localization and on cell division. (a) Phase-contrast images of C. difficile cells of *cwlA* + P*_tet_ empty*, *cwlA* + P*_tet_ cwlA*-HA, *cwlA* + P*_tet_ cwlA-T405A*-HA, and *cwlA* + P*_tet_ cwlA-T405D*-HA strains. Scale bar, 5 μm. (b) Scatterplots showing the distribution of cell length for each strain. Two-sided Mann-Whitney *U* tests (****, *P < *0.0001; **, *P*< 0.01; ns = 0.9143); 190 (WT + P*_tet_ empty*), 230 (*cwlA* + P*_tet_ empty*), 146 (*cwlA* + P*_tet_ cwlA*-HA), 269 (*cwlA* + P*_tet_ T405A*-HA), and 167 (*cwlA* + P*_tet_ T405D*-HA) cells were counted. (c) Percentage of cells harboring a chaining phenotype. *n* indicates the number of cells counted per strain in a single representative experiment. (d, upper) Western blots showing the levels of CwlA, CwlA-T405A, and CwlA-T405D proteins in the different cell fractions: cytoplasm (Cyto), membrane (Mb), and cell wall (CW). Cwp66 was used as a fractionation control. These proteins were detected using anti-HA and anti-Cwp66 antibodies, respectively. (Lower) Quantification of CwlA signals by densitometry. The CwlA value of the cytoplasm fraction was arbitrarily set to 100, and asterisks indicate statistically significant differences. From left to right (cytoplasm), *P = *0.291 and *P = *0178 (ns); from left to right (CW fraction), *P = *0.028 and *P = *0.010 (*, *P < *0.05). (e, upper) Western blots showing the levels of CwlA-HA in the different cell fractions of STK and STP mutants for cytoplasm (Cyto) and cell wall (CW). (Lower) Quantification of signals by densitometry. From left to right (cytoplasm), *P = *0.126, 0.070, 0.265, and 0.478 (ns); from left to right (CW fraction), *P = *0.000011 (****, *P < *0.0001), 0.275 (ns), 0.014, and 0.44 (*, *P < *0.05). Average values and standard errors of the means are calculated from 4 independent experiments.

To study the role of the T405 phosphorylation, we expressed in the *cwlA* mutant genes encoding CwlA, CwlA-T405A, or CwlA-T405D fused to a hemagglutinin (HA) tag under the control of the inducible P*_tet_* promoter. Cells were grown for 3 h before adding 50 ng/ml ATc inducer for 2 h. Under these conditions, we observed that the level of overexpression of *cwlA* was only 2-fold, as determined by qRT-PCR (Fig. S7a). Cell length and separation defect were restored in cells expressing a WT copy of *cwlA* (Fig. 6a to c and Fig. S7b and c) or *cwlA-T405A*. In contrast, cells expressing *cwlA-T405D* retained a defect in cell length and in cell separation, similar to cells carrying an empty plasmid (Fig. 6a to c). We then analyzed by Western blotting the distribution of CwlA-HA in the different cell fractions (cytoplasm, membrane, and CW). As a fractionation control, we used antibodies raised against Cwp66, a CW-associated protein of C. difficile (42–44). Cwp66 was mainly detected in the CW fraction (Fig. 6d), as expected. Using an anti-HA antibody, we detected similar amounts of CwlA-HA in the cytoplasm and in the membrane for the three strains. In contrast, compared to the CwlA protein, a slight but significant reduction of the amount of CwlA-T405D and an increase of CwlA-T405A were detected in the CW (Fig. 6d). These results suggest that phosphorylation plays a role in controlling the export of the CwlA PGH.

10.1128/mBio.00519-21.7 FIG S7 Complementation of *cwlA* mutant with pDIA6103-P*_tet_ cwlA*-HA plasmid. (a) qRT-PCR analysis estimates the level of *cwlA* expression in the *cwlA*::*erm* mutant and the complemented strains compared to the WT strain. T3-20 means 3 h of growth followed by 2 h of induction at 20 ng/ml, and T3-50 means 3 h of growth followed by 2 h of induction at 50 ng/ml. The result presented is the mean from the data obtained with 4 independent RNA samples. (b) Phase-contrast images of WT, *cwlA*, and *cwlA*+ P*_tet_ cwlA*-HA cells after 5 h of growth in TY. Induction was performed with 50 ng/ml ATc from the beginning of inoculation (T0) or after 3 h of growth (T3). Scare bar, 5 μm. (c) Scatter plots showing cell length, with the median and SD of each distribution indicated by a black line (upper) and percentage of cells harboring a chaining phenotype (lower). *n* indicates the number of cells counted per strain in a single representative experiment. The images and *n* values are representatives of experiments performed in triplicate. Download FIG S7, PDF file, 1.5 MB. Copyright © 2021 Garcia-Garcia et al. 2021 Garcia-Garcia et al. https://creativecommons.org/licenses/by/4.0/ This content is distributed under the terms of the Creative Commons Attribution 4.0 International license.

### PrkC and CD2148 influence the abundance of cell wall-associated CwlA.

To confirm the influence of phosphorylation on the export of CwlA, we investigated the cellular location of CwlA-HA in strains expressing P*_tet_ cwlA-*HA and inactivated for the STKs or STP. We detected CwlA-HA in both the cytoplasm and the CW in all the tested strains. While the amounts of CwlA-HA detected in the cytoplasm and the level of Cwp66 in the CW were stable in all strains, we observed variations of the abundance of CwlA in the CW fraction (Fig. 6e). Compared to the WT strain, CwlA-HA levels increased approximately 2.5- and 2-fold in the CW of the Δ*prkC* mutant and the Δ*prkC CD2148* double mutant, respectively. In contrast, the amount of CwlA-HA was unchanged in the CW of the *stp* mutant and significantly reduced in the CW of the *CD2148* mutant (Fig. 6e). Reduced CwlA levels found in the CW of the *CD2148* mutant might be due to the shedding of this PGH into the supernatant. To test this, we determined the levels of CwlA-HA in the supernatant of the different strains. No CwlA-HA was detected in the supernatant of the *CD2148* mutant. Altogether, these results revealed an inverse correlation between the level of phosphorylation of CwlA (Fig. 5a) and its presence in the CW (Fig. 6e), supporting a model where the ability of CwlA to reach the CW and, thus, exert PG hydrolysis is dependent on its phosphorylation level.

### Overexpression of *cwlA* restores the cell separation defect in *CD2148* cells.

**FIG 7:**
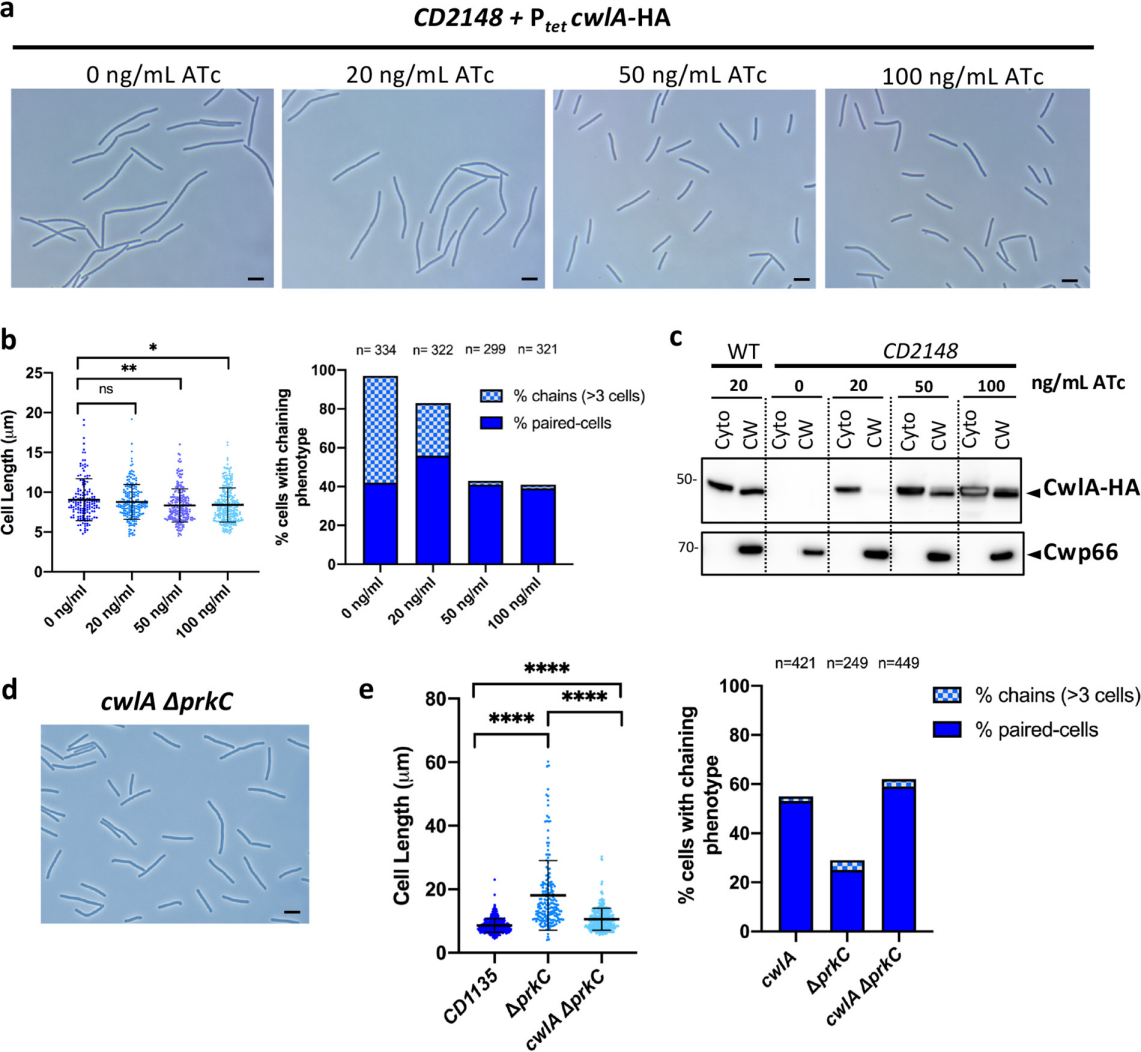
Overproduction or lack of CwlA restored the phenotypes of *CD2148* and Δ*prkC* mutants, respectively. (a) Phase-contrast images of exponentially growing *CD2148* cells expressing *cwlA*-HA under the control of a P*_tet_* promoter at different ATc concentrations (0, 20, 50, and 100 ng/ml). Scale bar, 5 μm. (b, left) Scatterplots showing cell length, with the median and SD of each distribution indicated by a black line. *P* values were determined by two-sided Mann-Whitney *U* tests. *P* = 0.7146 (ns), 0.0074 (**, *P < *0.01), and 0.0116 (*, *P < *0.05); 152 (0 ng/ml), 197 (20 ng/ml), 236 (50 ng/ml) and 287 (100 ng/ml) cells were counted. (Right) Percentage of cells harboring a chaining phenotype. *n* indicates the number of cells counted per strain in a single representative experiment. (c) Western blots showing the levels of CwlA-HA in the cytoplasm (Cyto) and cell wall (CW) of *CD2148* mutant in the presence of increasing concentrations of ATc. A sample corresponding to the WT strain overexpressing *cwlA* in the presence of 20 ng/ml of ATc was used as a control. (d) Phase-contrast images of the *cwlA* Δ*prkC* double mutant. Scale bar, 5 μm. (e) Scatterplots showing cell length (left) and percentage of cells harboring a chaining phenotype (right). Two-sided Mann-Whitney *U* tests (****, *P < *0.0001); 431 (*cwlA*), 183 (Δ*prkC*), and 329 (*cwlA* Δ*prkC*) cells were counted.

Cell division defects observed in the *CD2148* mutant are probably due to the decreased level of CwlA in the CW (Fig. 6e). We next investigated whether *cwlA* overexpression in the *CD2148* mutant could restore a WT phenotype. The *CD2148* mutant carrying P*_tet_ cwlA*-HA on a plasmid was grown for 3 h before inducing the expression of *cwlA* with a range of ATc concentrations. In the absence of induction, 97% of the cells exhibited a chaining phenotype, 55% with 3 or more unseparated cells. After induction with 20 ng/ml ATc, 83% of cells exhibited separation defects, with 27% as chains of at least 3 cells. However, in the presence of 50 or 100 ng/ml ATc, we observed a slight but significant reduction of cell length and 41 or 39% of paired cells, respectively. In addition, only 2% of chains of more than 3 cells was detected instead of 55% for the control in the absence of induction (Fig. 7a and b). To correlate these results to the accumulation of CwlA in the CW, CwlA levels were determined by Western blotting. In the presence of 20 ng/ml ATc, CwlA was barely detected in the CW, in agreement with the phenotype observed under this condition. However, the level of CwlA in the CW increased substantially when CwlA expression was induced with 50 or 100 ng/ml ATc (Fig. 7c). In the presence of the highest concentration of ATc, the amount of CwlA reached the level detected in the CW of the WT strain carrying P*_tet_*-*cwlA* in the presence of 20 ng/ml of ATc (Fig. 7c). These amounts of CwlA in the CW were sufficient to partially restore a WT phenotype. Overexpression of *cwlA* compensates for the absence of CD2148. This might be due to an increase of the pool of nonphosphorylated CwlA present in the CW restoring cell separation.

### Cell elongation and antimicrobial susceptibility are restored in the *cwlA ΔprkC* double mutant.

The cell division and morphology defects observed in the Δ*prkC* mutant could also be linked to the level of the PGH CwlA in the CW. To determine if the high level of CwlA in the CW of the Δ*prkC* mutant triggers cell elongation, we constructed a *cwlA* Δ*prkC* double mutant. Phase-contrast microscopy analysis of *cwlA* Δ*prkC* mutant cells revealed a significant reduction in cell length (10.5 ± 3.4 μm) compared to that of Δ*prkC* cells (18.1 ± 10.9 μm) and a slight but significant increase in size compared to the *cwlA* mutant (8.6 ± 2.1 μm) (Fig. 7d and e). The *cwlA* Δ*prkC* double mutant also presented a chaining phenotype similar to that of the *cwlA* mutant (Fig. 7e, right). These results indicate that elongation of cells in the Δ*prkC* mutant is mainly due to the accumulation of CwlA in the CW. CwlA plays an important role in the metabolism of PG. We previously reported the high susceptibility of the Δ*prkC* mutant to antimicrobial compounds targeting CW (29). Here, we compared the level of susceptibility of the mutants inactivated for *prkC*, *CD2148*, *stp*, and *cwlA* as well as the *cwlA prkC* double mutant to two cephalosporins (cefoxitine and ceftazidime), teicoplanin, and bacitracin (Table 2). MICs of the *stp*, *CD2148*, or *cwlA* mutants were comparable to that of the WT strain for the compounds tested. Interestingly, inactivation of *cwlA* into the Δ*prkC* mutant restored resistance to the tested compounds to the WT level. Thus, these data suggest that the susceptibly of the Δ*prkC* mutant to the CW-targeting antimicrobial compounds is caused by the accumulation of CwlA in the CW.

**TABLE 2:** MICs of the mutants used in this study for antibiotics targeting CW

Antibiotics	MIC^*a*^ (μg/ml)					
	630 *Δerm*	*ΔprkC*	*Δstp*	*CD2148*	*cwlA*	*cwlA ΔprkC*
Cephalosporins						
Cefoxitin	>256	48	>256	>256	>256	>256
Ceftazidime	64	3	64	96	96	64
Other cell wall						
Teicoplanin	0.12	<0.016			0.12	0.12
Bacitracin	>256	8	>256	>256	>256	>256

a MICs are reported as the medians from three independent experiments performed in BHI broth at 37°C for 24 h.

## DISCUSSION

In this study, we characterized a new PGH of C. difficile, CwlA, that cleaves the PG and plays a role in cell division. In addition, we identified the STK PrkC as a direct regulator for this hydrolase by modulating CwlA localization and access to the CW, highlighting a novel regulatory mechanism to control CW hydrolysis by phosphorylation.

When bacteria divide, they form a septum, and each daughter cell contains a membrane with a layer of PG that is shared between them. To separate the cells and complete the cell division process, the shared PG must be partially hydrolyzed by dedicated enzymes (45). CwlA belongs to the NlpC/P60 family, a major class of CW-degrading enzymes that typically function as endopeptidases and hydrolyze γ-d-glutamyl-*meso*-DAP or NAM-l-alanine linkages (33). Here, we demonstrated that CwlA cleaves the cross-linked PG at the level of stem peptides. We identified the first PGH involved in cell division in C. difficile allowing separation of daughter cells. Accordingly, around 50% of cells were found as a chain of paired cells in a mutant lacking *cwlA.* Even if phenotypes are sometimes difficult to observe with a single PG hydrolase gene knockout, probably due to functional redundancy (5), mutants lacking splitting hydrolases can fail to properly divide and form chains of unseparated cells, as observed for the *cwlA* mutant. An *atl* mutant of S. aureus and a *lytB* mutant of S. pneumoniae form clusters of nonseparated cells. These hydrolases are localized at the sites of cell division (46, 47), as observed for CwlA. In B. subtilis, LytE, LytF, CwlS, or CwlO hydrolyzes the linkage of γ-d-glutamyl-*meso*-DAP in PG (48–50). LytE and CwlO break the PG along the lateral CW to support cell elongation, while LytF and CwlS are implicated in cell separation (50–52). Inactivation of either *cwlO* or *lytE* produces shorter cells, and a double mutant is lethal (53). In contrast to LytF, LytE, and CwlS, which contain several LysM PG-binding domains, CwlA possesses a less characterized SH3 PG-binding domain (54). Recently, it has been shown that the SH3 domain of lysostaphin recognizes the pentaglycine cross-bridges present in staphylococcal PG, positioning the enzyme to cleave the cross-bridges (55). However, these cross-bridges are absent from the PG of C. difficile (56), indicating a different SH3-binding motif. Interestingly, a recent biochemical and structural characterization of the C. difficile Acd24020 protein revealed that the SH3_3 domains of this autolysin could recognize the 3 to 4 cross-linking structures (32), and this might also be the case for the CwlA endopeptidase. Overproduction of highly active PGHs usually results in cell lysis (34). Surprisingly, we observed that overexpression of *cwlA* leads to cell elongation. The elongation phenotype that we observed is associated with the overproduction of the SH3_3 domains, which could impair the access of other PGHs to PG. Interestingly, C. difficile possesses three other NlpC/P60 endopeptidases associated with one to three SH3_3 domains (57), including the recently characterized Acd24020 (CD2402) (Fig. S8) that may have redundant functions with CwlA. A fourth PGH, Acd (CD1304), that hydrolyses peptidoglycan bonds between NAG and NAM, is associated with four SH3 domains (58).

10.1128/mBio.00519-21.8 FIG S8 Predicted PG-degrading enzymes associated with SH3 domains identified in C. difficile 630. Schematic representation of PG-degrading enzymes containing different numbers of SH3 domains (PF08239). Download FIG S8, PDF file, 0.08 MB. Copyright © 2021 Garcia-Garcia et al. 2021 Garcia-Garcia et al. https://creativecommons.org/licenses/by/4.0/ This content is distributed under the terms of the Creative Commons Attribution 4.0 International license.

Proper PG hydrolysis is essential for CW biogenesis. However, due to their destructive potential, PGH activity must be tightly regulated to ensure that hydrolases act when and where they should in coordination with PG synthesis to prevent CW damage (3, 34, 59). In this study, we uncovered a novel and original mechanism for PG hydrolysis control by STK-dependent phosphorylation. We demonstrated that CwlA is specifically phosphorylated at T405 *in vivo* by the PASTA-STK PrkC. Further *in vitro* phosphorylation using the purified kinases PrkC and CD2148 confirmed the T405 phosphorylation by PrkC. In other Gram-positive bacteria, PASTA-STKs control the expression of endopeptidase genes or other genes involved in CW metabolism through transcriptional regulators like WalR-WalK or GraR. PrkC of B. subtilis phosphorylates WalR, which controls *lytE* and *cwlO* genes, encoding endopeptidases in early stationary phase (8, 60). StkP in S. pneumoniae also has been proposed to function in concert with WalK through protein-protein interaction (61). PASTA-STKs also phosphorylate enzymes involved in PG metabolism, such as Glm or Mur enzymes and the flippase MviM (20, 24, 39, 62–64). Interestingly, the amidase CwlM is phosphorylated by the STK PknB in M. tuberculosis. However, in contrast to CwlA, which is exported to the CW, CwlM remains in the cytoplasm, and its phosphorylation stimulates the catalytic activity of MurA, the first enzyme in the PG precursor synthesis pathway (65). Finally, several proteins involved in cell division, such as DivIVA, MapZ, GspB, and FtsZ, are also phosphorylated by STKs in B. subtilis, S. pneumoniae, or S. aureus (22–24, 66, 67).

**FIG 8:**
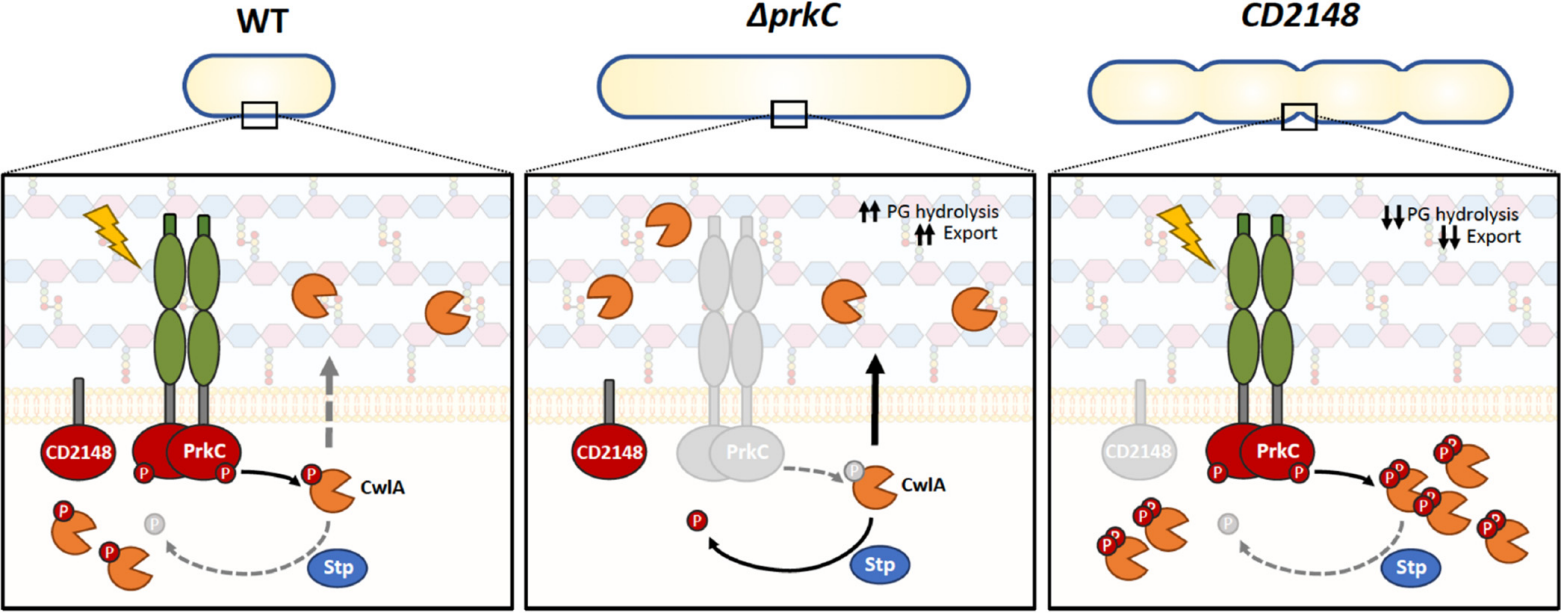
Model for STK-dependent regulation of the endopeptidase CwlA. PrkC could sense extracellular signals generated during PG synthesis (muropeptides, lipid II, or other) through its PASTA domains. (Left) Active PrkC phosphorylates CwlA (orange scissors), inhibiting its export. Hence, the phosphatase STP dephosphorylates the PrkC-target CwlA, and this protein is exported to the CW, increasing PG hydrolysis when required. (Middle) In a Δ*prkC* mutant, the nonphosphorylated form of CwlA is more efficiently exported to the CW, and a high level of CwlA triggers cell elongation. (Right) In a *CD2148* mutant, PrkC highly phosphorylates CwlA. As a consequence, this endopeptidase is less exported, resulting in a cell separation defect.

We evidenced that CwlA phosphorylation by PrkC does not affect its catalytic function but rather reduces its presence in the CW, likely through inhibition of its export. Therefore, in this work, we propose a novel mechanism of control of CW homeostasis by the STKs. In our model (Fig. 8), the homeostatic control of CwlA ensures that growing cells maintain a defined amount of hydrolase activity for cytokinesis. We propose that modulation of the export of PGHs by the STK-dependent signaling pathway is one mechanism of cell adaptation during cell division. As observed in other firmicutes (19–21), PrkC of C. difficile could sense extracellular signals generated during PG synthesis (muropeptides, lipid II, or other) via its PASTA domains. In the WT strain, appropriate hydrolytic activity is controlled by PrkC and STP, which can phosphorylate and dephosphorylate CwlA, respectively, limiting or increasing its availability at the cell surface. In the Δ*prkC* mutant, the nonphosphorylated form of CwlA increases and could be more efficiently exported to the CW, triggering cell elongation. In the *CD2148* mutant, PrkC could phosphorylate CwlA more efficiently and on multiple sites, as observed *in vivo* (Fig. S6a), limiting the presence of CwlA in the CW and resulting in a cell separation defect (Fig. 8). In the *CD2148* mutant, the overexpression of *cwlA* restores a WT phenotype by increasing the amount of CwlA able to reach the CW. However, further studies are required to understand the complex regulatory role of CD2148. One hypothesis is that CD2148 interacts at the septum of the cells either directly with PrkC or indirectly through proteins of the divisome. These interactions could stimulate PrkC activity or control the choice of its substrates. A second hypothesis is that CD2148 has a phosphatase activity, thereby dephosphorylating CwlA to increase its export.

## MATERIALS AND METHODS

### Bacterial strains and growth conditions.

Bacterial strains and plasmids used in this study are listed in Table S1 in the supplemental material. C. difficile strains were routinely grown at 37°C in an anaerobic environment (90% N_2_, 5% CO_2_, and 5% H_2_) in TY (30 g/liter Bacto tryptone, 20 g/liter yeast extract, pH 7.4) or in brain heart infusion (BHI; Difco). When necessary, C. difficile culture media were supplemented with cefoxitin (Cfx; 25 μg/liter), cycloserine (Ccs; 250 μg/liter), thiamphenicol (Tm; 7.5 μg/liter), and erythromycin (Erm; 5 μg/liter). E. coli strains were cultured at 37°C in LB broth containing chloramphenicol (25 μg/liter), kanamycin (25 μg/ml), or ampicillin (100 μg/liter) when necessary. ATc was used to induce the expression of the *cwlA* gene from the P*_tet_* promoter of pDIA6103 (68). To determine MICs, cultures of C. difficile strains (optical density at 600 nm [OD_600_] of 0.3) were plated on BHI agar plates, and the MICs were determined by Etest (bioMérieux) after 24 h of incubation at 37°C.

10.1128/mBio.00519-21.9 TABLE S1 Strains and plasmids used in this study. Download Table S1, DOCX file, 0.02 MB. Copyright © 2021 Garcia-Garcia et al. 2021 Garcia-Garcia et al. https://creativecommons.org/licenses/by/4.0/ This content is distributed under the terms of the Creative Commons Attribution 4.0 International license.

### Construction of C. difficile strains and plasmids.

All routine plasmid constructions were carried out using standard procedures. To generate the *cwlA*::*erm* and *CD2148*::*erm* mutants, the ClosTron system was used as previously described (69). Briefly, primers designed to retarget the group II intron on pMTL007 to *cwlA* and *CD2148* were used with the EBS universal primer and intron template DNA to generate a 353-bp DNA fragment for each gene by overlap PCR (Table S2). The PCR products were cloned into the HindIII and BsrGI restriction sites of pMTL007, and the sequence of the insertions was verified by sequencing (Table S2). Plasmids pMTL007::*cwlA*-1164s and pMTL::*CD2148*-302a retargeted the group II intron for insertion into *cwlA* and *CD2148* after the 1,164th and the 302th nucleotide in the coding sequence, respectively. E. coli HB101(RP4) strains containing these plasmids were transferred by conjugation into the C. difficile 630Δ*erm* strain. Transconjugants selected on BHI plates containing Cfx, Ccs, and Tm were plated on BHI agar containing Erm. Erm-resistant C. difficile colonies corresponded to plasmid loss and insertion of the group II intron into the chromosome (69). The insertion of introns into the target gene was verified by PCR. A *Δstp* knockout mutant was generated using the *codA*-mediated allele exchange method (ACE) (29, 70, 71). Fragments (1 kb) located up- and downstream of *stp* were amplified from 630Δ*erm* genomic DNA (Table S2). Purified fragments were then introduced into the pMTLSC7315 ΔMCS by Gilson assembly master mix (Biolabs), giving the plasmid pDIA6464 (Table S1). E. coli HB101(RP4) containing pDIA6464 was mated with the C. difficile 630Δ*erm* strain. After conjugation, faster-growing single-crossover integrants were isolated by serially restreaking on BHI plates supplemented with Cfx and Tm. Double crossover events were obtained by restreaking single crossover integrants on C. difficile minimal medium plates supplemented with fluorocytosine (50 μg·ml^−1^).

10.1128/mBio.00519-21.10 TABLE S2 Oligonucleotides used in this study. Download Table S2, DOCX file, 0.02 MB. Copyright © 2021 Garcia-Garcia et al. 2021 Garcia-Garcia et al. https://creativecommons.org/licenses/by/4.0/ This content is distributed under the terms of the Creative Commons Attribution 4.0 International license.

To complement the *cwlA*::*erm* and *CD2148*::*erm* mutants, the *cwlA* and *CD2148* genes, with their own promoters, were amplified by PCR (72). Fragments were introduced into pMTL84121 using the Gibson assembly master mix. Using E. coli HB101(RP4) as a donor, the resulting plasmids were introduced by conjugation into the *cwlA*::*erm* or *CD2148*::*erm* mutant. To construct a plasmid expressing *cwlA* or *stp* under the control of a P*_tet_* promoter inducible by ATc, PCR fragments containing complete genes were amplified and cloned into pDIA6103. For truncated *cwlA* lacking the catalytic NlpC/P60 domain or the SH3 domains, plasmid pDIA6103-P*_tet_*-*cwlA* was amplified by inverse PCR. The PCR product was then digested by DpnI to remove the plasmid template, phosphorylated by T4 polynucleotide kinase, and ligated by T4 ligase to recircularize the plasmid. The same strategy was used to construct the translational fusion coding for a CwlA-HA-tagged protein and to introduce a point mutation into the *cwlA* gene (threonine at position 405 replaced by an alanine [T405A] or a glutamate [T405D]). All generated plasmids and primers are listed in Tables S1 and S2. pDIA7128 carrying a translational *cwlA*-SNAP fusion was obtained by Gibson assembly. We amplified the SNAP coding sequence fused to a linker in 5′ orientation (GGATCCGCAGCTGCT) using pFT58 as a template, and pDIA6912 (pMTL84121-*cwlA*) was amplified by inverse PCR (Table S2). These plasmids were transferred into C. difficile strains by conjugation.

### RNA extraction and quantitative RT-PCR analysis.

Cells were harvested after 5 h of growth in the TY medium with antibiotics and ATc when required. The culture pellets were resuspended in the RNApro solution (MP Biomedicals), and RNA was extracted using the FastRNA Pro blue kit. cDNA synthesis and real-time quantitative PCR were performed as previously described (73). In each sample, the quantity of cDNAs of a gene was normalized to the quantity of cDNAs of the *DNA pol III* and *gyrA* genes. The relative change in gene expression was recorded as the ratio of normalized target concentrations (the threshold cycle [ΔΔ*C_T_*] method).

### CwlA homology model.

The CwlA 3D homology model was generated using the Phyre2 server (41) (http://www.sbg.bio.ic.ac.uk/phyre2/) based on the alignment with c6biqA, c3npfB, and c3h41A (83% of the sequence modeled at >90% accuracy) and displayed in EzMol 2.1 (74) (http://www.sbg.bio.ic.ac.uk/ezmol/). A sequence alignment of *cwlA* with conserved regions of NlpC/P60 domains of B. cereus YkfC and B. subtilis YkfC, LytF, LytE, and CwlS was generated using Clustal Omega (http://www.ebi.ac.uk/Tools/msa/clustalo/) and ESPript 3.0 (75) (http://espript.ibcp.fr/ESPript/ESPript/) to identify the catalytic residues in CwlA belonging to the endopeptidase NlpC/P60 family of proteins.

### Protein synthesis and purification.

To purify the kinase domain (KD) of PrkC or CD2148, DNA sequence encoding the cytosolic part of each protein was PCR amplified from genomic DNA using the primer pairs SAT117/SAT118 and SAT119/SAT264, respectively. The region of *cwlA* coding for the CW-associated hydrolase residues 32 to 432 (the N-terminal amino acids corresponding to the signal peptide were omitted) was amplified using the oligonucleotides SAT285 and SAT286. The PCR products digested with BamHI and KpnI were cloned into the expression vector pQE30, giving plasmids pQE30-*prkC-*KD, pQE30-*CD2148*-KD, and pQE30-*cwlA*. These plasmids carried genes encoding proteins fused to a His_6_ tag expressed under the control of an IPTG (isopropylthiogalactoside)-inducible promoter. His_6_-tagged proteins were produced in E. coli strain M15pRep4. Cultures were grown at 37°C to an OD_600_ of 0.4 and the genes induced for 3 h in the presence of 1 mM IPTG. Cells were disrupted by sonication, and N terminus His_6_-tagged proteins were purified on Ni-NTA columns (Qiagen) according to the manufacturer’s instruction, desalted with PD-10 columns (GE-Healthcare), and stored at −20°C in a buffer containing 50 mM Tris-Cl, pH 7.5, 100 mM NaCl, and 10% glycerol. Protein concentrations were estimated using the Bradford assay (Bio-Rad) with bovine serum albumin (BSA) as the standard.

### *In vitro* phosphorylation assay and Phos-Tag.

His_6_-CwlA (10 μM) was incubated alone or in combination with His_6_-PrkC-KD or His_6_-CD2148-KD (1 μM) in kinase buffer (50 mM Tris, pH 7.5, 5 mM MgCl_2_). The reaction was initiated by the addition of 5 mM ATP, followed by incubation at 37°C for 1.5 h. Reactions were stopped with the addition of SDS-Laemmli sample buffer, and proteins were subjected to Phos-Tag or SDS-PAGE. Phos-Tag is based on the functional molecule Phos-Tag that captures phosphate groups (-PO_3_^2^) (37, 40). Two different methods were performed according to the manufacturer’s instructions: Phos-Tag fluorescence and Phos-Tag acrylamide. For Phos-Tag fluorescence, SDS-PAGE gels were incubated in a solution containing Phos-Tag (Phos-Tag phosphoprotein gel stain; ABP Biosciences) and then washed in Phos-Tag Phosphoprotein destain solution (ABP Biosciences). Phosphorylated proteins specifically stained were detected using a fluorescence imaging scanner. For Phos-Tag acrylamide, which is an electrophoresis technique capable of separating phosphorylated and nonphosphorylated forms based on phosphorylation levels, proteins were run on a 12% SDS-PAGE supplemented with 50 μM Phos-Tag acrylamide (AAL-107; Wako) and 100 μM MnCl_2_. After running, the gel was stained with Coomassie blue.

### In-gel and FASP-based digestion.

His_6_-PrkC was used to phosphorylate His_6_-CwlA *in vitro* as described above. The protein mixture was separated by SDS-PAGE and stained with Coomassie blue. The band corresponding to His_6_-CwlA was excised and subjected to tryptic digestion as previously described (76). Resulting peptides were dried in a Speed-Vac and resuspended in 2% acetonitrile (ACN), 0.1% formic acid (FA) prior to liquid chromatography-tandem MS (LC-MS/MS) analysis.

For FASP-based digestion, bacterial pellets were resuspended in 100 mM ammonium bicarbonate (ABC), 50 mM dithiothreitol, 4% SDS, 1% DNase I, 1× protease, and phosphatase inhibitors and disrupted by ultrasonic cavitation. Protein digestion was based on the FASP procedure using 30K Amicon Ultra-4 filtration devices (Millipore). Briefly, 4 mg protein lysate was concentrated into the filtration device at 4,500 × *g* for 20 min and diluted with 2 ml of exchange buffer (EB; 100 mM ABC, 8 M urea). This step was repeated 3 times before adding 1 ml EB containing 5 mM Tris(2-carboxyethyl)phosphine hydrochloride, 30 mM chloroacetamide, 0.3% Benzonase, 0.1% DNase I, 1 mM MgCl_2_ during 1.5 h at room temperature. After one replacement of buffer with EB, the resulting concentrate was washed by 3 steps with 50 mM ABC. After overnight incubation with sequencing-grade modified trypsin (Promega) at an enzyme/protein ratio of 1:100, peptides were collected by centrifugation of the filter.

### Phosphoenrichment.

Resulting peptides were desalted with Sep-Pak plus C_18_ cartridges (Waters) and eluted with 80% ACN, 0.1% heptafluorobutyric acid (HFBA; Sigma-Aldrich) and then adjusted at 6% HFBA. TiO_2_ beads (Sachtopore NP beads; 5 μM, 300 Å; Huntsman) were resuspended at 20 mg/ml in 30% ACN, 0.1% HFBA during 1 h and activated for 15 min with 80% ACN, 6% HFBA. Peptide solution was incubated for 30 min at room temperature with TiO_2_ (10:1 bead to peptide). Two washes with the same buffer and one with 50% ACN, 0.1% HFBA were performed before an elution with 10% NH_4_OH. pH was neutralized with 20% FA, and enriched peptides were freeze-dried. Finally, phosphopeptides were desalted by stage-tip using a C_18_ Empore disc and dried in a Speed-Vac. Peptides were resuspended in 2% ACN, 0.1% FA prior to LC-MS/MS analysis.

### LC-MS/MS analysis.

Tryptic peptides from in-gel digestion were analyzed on a Q Exactive plus instrument (Thermo Fisher Scientific) and phosphoenrichment peptides on a Q Exactive HF instrument (Thermo Fisher Scientific), both instruments coupled with an EASY nLC 1200 chromatography system (Thermo Fisher Scientific). Sample was loaded on an in-house packed 25-cm (for in-gel digestion) and 53-cm (phosphoenrichment) nano-HPLC column with C_18_ resin (1.9-μm particles, 100-Å pore size; Reprosil-Pur Basic C18-HD resin; Maisch GmbH, Ammerbuch-Entringen, Germany) after an equilibration step in 100% solvent A (H_2_O, 0.1% FA). Peptides were first eluted using a 2% to 5% gradient of solvent B (ACN, 0.1% FA) during 5 min, a 5% to 10% gradient during 20 min, a 10% to 30% gradient during 70 min, and finally a 30% to 60% gradient during 20 min, all at 300 nl·min^−1^ flow rates. The instrument method was set up in the data-dependent acquisition mode. After a survey scan in the Orbitrap (resolution, 70,000 and 60,000), the 10 most intense precursor ions were selected for HCD fragmentation with normalized collision energy set to 27 and 28. Charge state screening was enabled, and precursors with unknown charge state or a charge state of 1, 7, 8, and >8 were excluded. Dynamic exclusion was enabled for 20 s and 30 s.

### Data processing for protein identification and quantification.

Raw files were searched MaxQuant software (77), version 1.5.3.8, with Andromeda (78) as a search engine against an internal C. difficile database containing 3,957 proteins (29), usual known mass spectrometry contaminants, and reversed sequences of all entries. Andromeda searches were performed choosing trypsin as the specific enzyme with a maximum number of three missed cleavages. Possible modifications included carbamidomethylation (Cys; fixed), oxidation (Met; variable), N-terminal acetylation (variable), and phospho (Ser, Thr, Tyr; variable). The mass tolerance in MS was set to 20 ppm for the first search, 4.5 ppm for the main search, and 20 ppm for the MS/MS. Maximum peptide charge was set to seven, and seven amino acids were required as the minimum peptide length.

The “match between runs” feature was applied for samples having the same experimental condition, with a maximal retention time window of 0.7 min. One peptide unique to the protein group was required for protein identification for the phosphoenrichment analysis. A false discovery rate (FDR) cutoff of 1% was applied at the peptide and protein levels. For in-gel analysis, we used the Phospho (STY) table to extract the intensity of each phosphopeptide. A normalization step based on the iBAQ of the protein in each sample was performed before relative quantification.

### Detection of PG-hydrolyzing activity and identification of hydrolytic specificity of CwlA.

PG samples were prepared from 600 ml of different strains of C. difficile grown in TY (OD_600_ of 1). PG-PSII was first purified as previously described (29) and used for the detection of the lytic activity of the purified enzymes by zymogram analysis. C. difficile purified PG was resuspended in distilled H_2_O, and the suspension was added to an SDS-polyacrylamide gel at a final concentration of 1 mg/ml. After electrophoresis, the gel was shaken at 37°C for 16 h in 50 ml of 25 mM Tris-HCl (pH 8.0) solution containing 1% (vol/vol) Triton X-100 to allow protein renaturation. Clear bands resulting from lytic activity were visualized after staining with 1% (wt/vol) methylene blue (Sigma)-0.01% (wt/vol) KOH and subsequent destaining with distilled water (57, 79). The PG was further separated from PSII by hydrofluoric acid treatment (29). To identify the hydrolytic specificity of CwlA, purified PG (0.75 mg) was incubated with 100 μg of purified His_6_-CwlA in 50 mM Tris-HCl, pH 8.0, overnight at 37°C. A control sample of PG was incubated without enzyme under the same conditions. After incubation, soluble and insoluble fractions were separated by centrifugation at 20,000 × *g* for 15 min. The insoluble fraction was further digested with mutanolysin from Streptomyces globisporus (Sigma) at 2,500 U·ml^−1^ for 19 h at 37°C in 25 mM sodium phosphate under shaking. The soluble muropeptides were reduced with sodium borohydride and analyzed by RP-HPLC and mass spectrometry as described previously (56).

### Phase-contrast, SNAP labeling, and fluorescence microscopy.

For phase-contrast microscopy, C. difficile strains were cultured for 5 h in TY (with antibiotics and inducers when needed) at 37°C. Cells were visualized using a Zeiss Axioskop microscope and analyzed using the software ImageJ and the plugin MicrobeJ (80) for quantitative analysis. For membrane staining, 500 μl of exponentially growing cells in TY was centrifuged and resuspended in 100 μl of phosphate-buffered saline (PBS) supplemented with the fluorescent membrane dye FM 4-64 (Molecular Probes, Invitrogen) at 1 μg/ml. Samples were incubated for 2 min in the dark and mounted on 1.2% agarose-coated glass slides. For SNAP labeling, strains were grown for 3 h in TY, and the expression of the SNAP*^Cd^*-*CD2148* fusions was induced with 50 ng/ml ATc for 2 h. The TMR-Star substrate (New England Biolabs) was added at 250 nM, and the mixture was incubated for 30 min in the dark under anaerobiosis. Cells were then collected by centrifugation, washed, and resuspended in PBS. Cell suspension (3 μl) was mounted on a 1.2% agarose pad. The images were taken with exposure times of 600 ms for autofluorescence and 800 ms for SNAP using a Nikon Eclipse TI-E microscope, 100× objective, and captured with a CoolSNAP HQ2 camera. The images were analyzed using ImageJ.

### TEM.

C. difficile strains were grown in TY at 37°C for 5 h. After centrifugation at 5,000 rpm for 10 min at 4°C, cell pellets were resuspended in 2% glutaraldehyde in 0.1 M cacodylate buffer and incubated for 1 h at room temperature. Cells pellets then were washed with 0.2 M sucrose in 0.1 M cacodylate buffer. Staining and examinations were done by the GABI-MIMA2 TEM platform at INRA, Jouy-en-Josas, France. Grids were examined with a Hitachi HT7700 electron microscope operated at 80 kV, and images were acquired with a charge-coupled device camera (AMT).

### Cell lysis, fractionation, and protein analysis.

We detected the production of a CwlA-HA-tagged protein by Western blotting using an antibody raised against the HA tag. Cellular fractions were extracted as previously described (71). Briefly, C. difficile cultures (10 ml) were harvested by centrifugation at 5,000 × *g* for 10 min at 4°C, and culture supernatants (Sn) were filtered through a 0.22-μm-pore-size filter and precipitated on ice with 10% trichloroacetic acid for 30 min (Sn) prior to SDS-PAGE. Pellets resuspended in phosphate-sucrose buffer (0.05 M HNa_2_PO_4_, pH 7.0, 0.5 M sucrose) to an OD_600_ of 40 were incubated at 37°C for 1 h in the presence of purified CD27L endolysin (30 μg/ml). The protoplasts were then recovered by centrifugation at 6,000 × *g* for 20 min at 4°C. Supernatants containing the CW fraction were removed, and the protoplast pellet was resuspended in phosphate buffer (0.05 M HNa_2_PO_4_, pH 7.0) containing 40 μg/ml DNase I at an OD_600_ of 40 and incubated at 37°C for 45 min. Lysates were harvested at 16,000 × *g* for 15 min at 4°C to separate supernatants containing the cytoplasmic (Cyto) fraction and the membrane pellet. For analysis by SDS-PAGE, an equal volume of 2× SDS sample buffer was added to protein samples. SDS-PAGE and Western immunoblotting were carried out using standard methods. Proteins were electrophoresed and transferred to polyvinylidene difluoride (PVDF) membranes. After blocking with nonfat milk in Tris-buffered saline-Tween 20 buffer, primary antibodies were added. The washed membranes were incubated with appropriate secondary antibodies coupled to horseradish peroxidase that were detected by an enhanced chemiluminescence system. Antibodies against the HA and SNAP epitopes were purchased from Osenses and New England BioLabs, respectively.

### Data availability.

The mass spectrometry proteomics data have been deposited with the ProteomeXchange Consortium (http://proteomecentral.proteomexchange.org) via the PRIDE partner (81) repository with the data set identifier PXD021541.
