# Supplementary material for: Ser/Thr Kinase-Dependent Phosphorylation of the Peptidoglycan Hydrolase CwlA Controls Its Export and Modulates Cell Division in Clostridioides difficile
Source: mBio. 2021 May 18;12(3):e00519-21. doi: 10.1128/mBio.00519-21 (PMC8262956; doi:10.1128/mBio.00519-21)
Supplement: FIG S1 [file mbio.00519-21-sf001.pdf]

Supplementary Figure 1

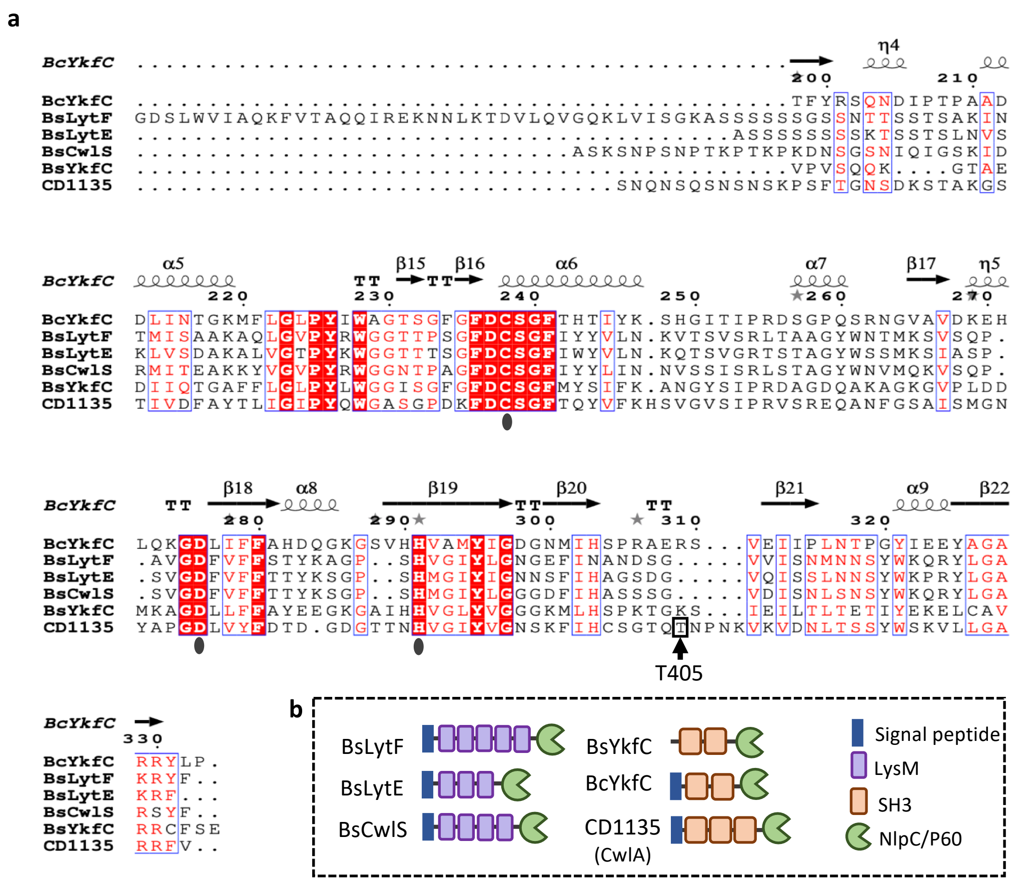

Supplementary Figure 1. CD1135 (CwlA) belongs to the NlpC/P60 family. a, Alignment of CD1135 with conserved regions of NlpC/P60 domains of *B. cereus* YkfC (BcYkfC), *B. subtilis* YkfC (BsYkfC), LytF (BsLytF), LytE (BsLytE) and CwlS (BsCwlS) contain the essential residues for catalysis (Cys, His, Asp; point black). Conserved residues are in red boxes and similar residues in red characters. **b,** Schematic representation of *B. subtilis* LytF, LytE, CwlS, YkfC, *B. cereus* YkfC and CD1135.
