# Supplementary material for: Ser/Thr Kinase-Dependent Phosphorylation of the Peptidoglycan Hydrolase CwlA Controls Its Export and Modulates Cell Division in Clostridioides difficile
Source: mBio. 2021 May 18;12(3):e00519-21. doi: 10.1128/mBio.00519-21 (PMC8262956; doi:10.1128/mBio.00519-21)
Supplement: FIG S2 [file mbio.00519-21-sf002.pdf]

Supplementary Figure 2

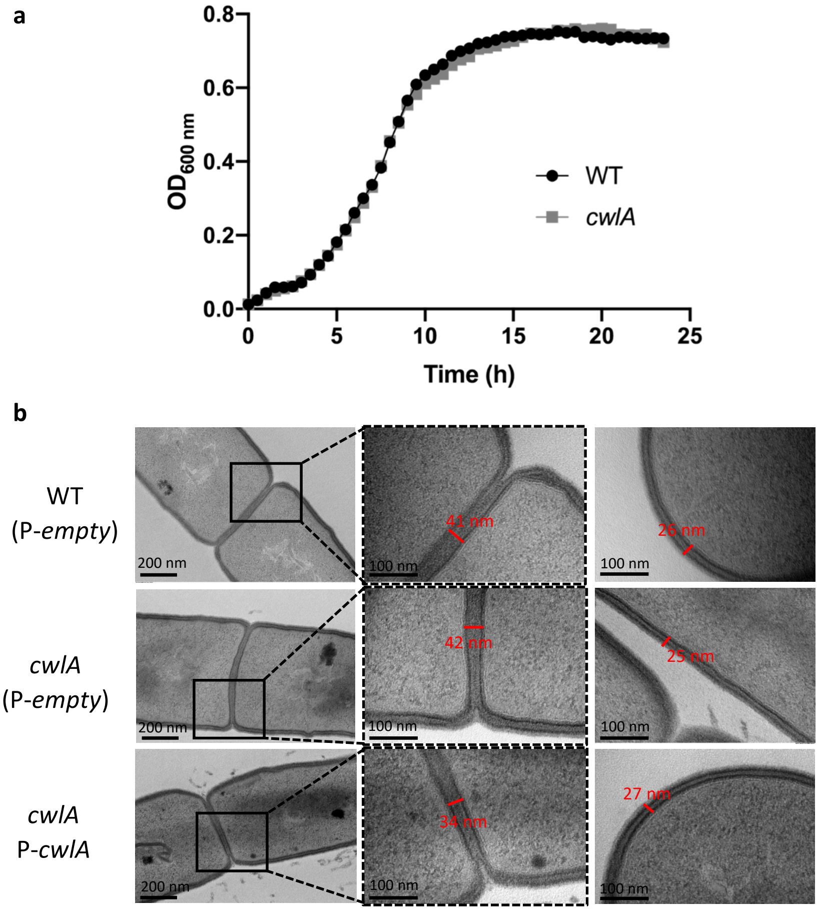

Supplementary Figure 2. Growth and TEM of *cwlA* mutant. **a,** Growth curves of *cwlA* mutant compared to WT strain in TY. **b,** Transmission electron microscopy showing septa thickness and cell wall width of 630Δ*erm* + P-*empty* (WT), *cwlA* + P-*empty* and *cwlA* + P-*cwlA*. Middle and right columns: higher magnifications (100 nm) of the division septa (highlighted by a black square) and cell wall, respectively.
