# Supplementary material for: Ser/Thr Kinase-Dependent Phosphorylation of the Peptidoglycan Hydrolase CwlA Controls Its Export and Modulates Cell Division in Clostridioides difficile
Source: mBio. 2021 May 18;12(3):e00519-21. doi: 10.1128/mBio.00519-21 (PMC8262956; doi:10.1128/mBio.00519-21)
Supplement: FIG S3 [file mbio.00519-21-sf003.pdf]

Supplementary Figure 3

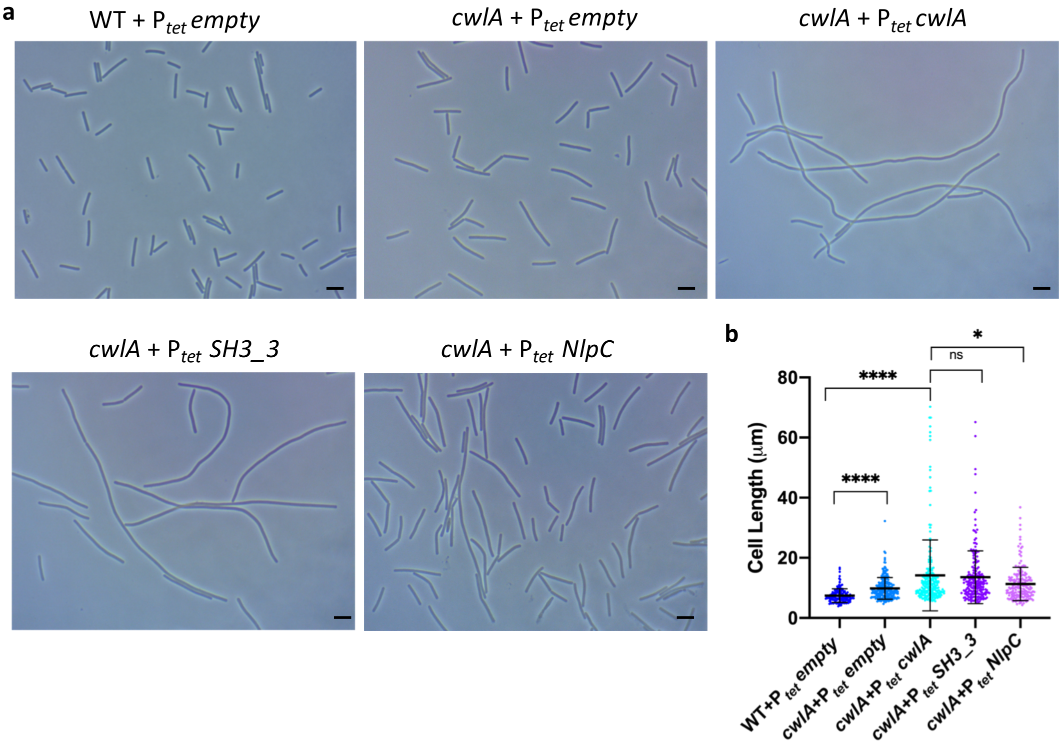

Supplementary Figure 3. Morphology of cells overexpressing *cwlA*. a, Phase contrast images of *C. difficile* cells of WT + P_{tet} *empty*, *cwlA* + P_{tet} *empty*, *cwlA* + P_{tet} *cwlA, cwlA* + P_{tet} *SH3_3* and *cwlA* + P_{tet} *NlpC*. Scare bar, 5 µm. **b**, Scatter plots showing the distribution of cell length. Two-sided Mann–Whitney *U* tests (*****P* < 0.0001; **P*<0.05), cells counted 148 (WT + P_{tet} *empty*), 223 (*cwlA* + P_{tet} *empty*), 219 (*cwlA* + P_{tet} *cwlA*), 208 (*cwlA* + P_{tet} *SH3_3*) and 180 (*cwlA* + P_{tet} *NlpC)* in a single representative experiment. Experiments were performed in triplicate.
