# Supplementary material for: Ser/Thr Kinase-Dependent Phosphorylation of the Peptidoglycan Hydrolase CwlA Controls Its Export and Modulates Cell Division in Clostridioides difficile
Source: mBio. 2021 May 18;12(3):e00519-21. doi: 10.1128/mBio.00519-21 (PMC8262956; doi:10.1128/mBio.00519-21)
Supplement: FIG S4 [file mbio.00519-21-sf004.pdf]

Supplementary Figure 4

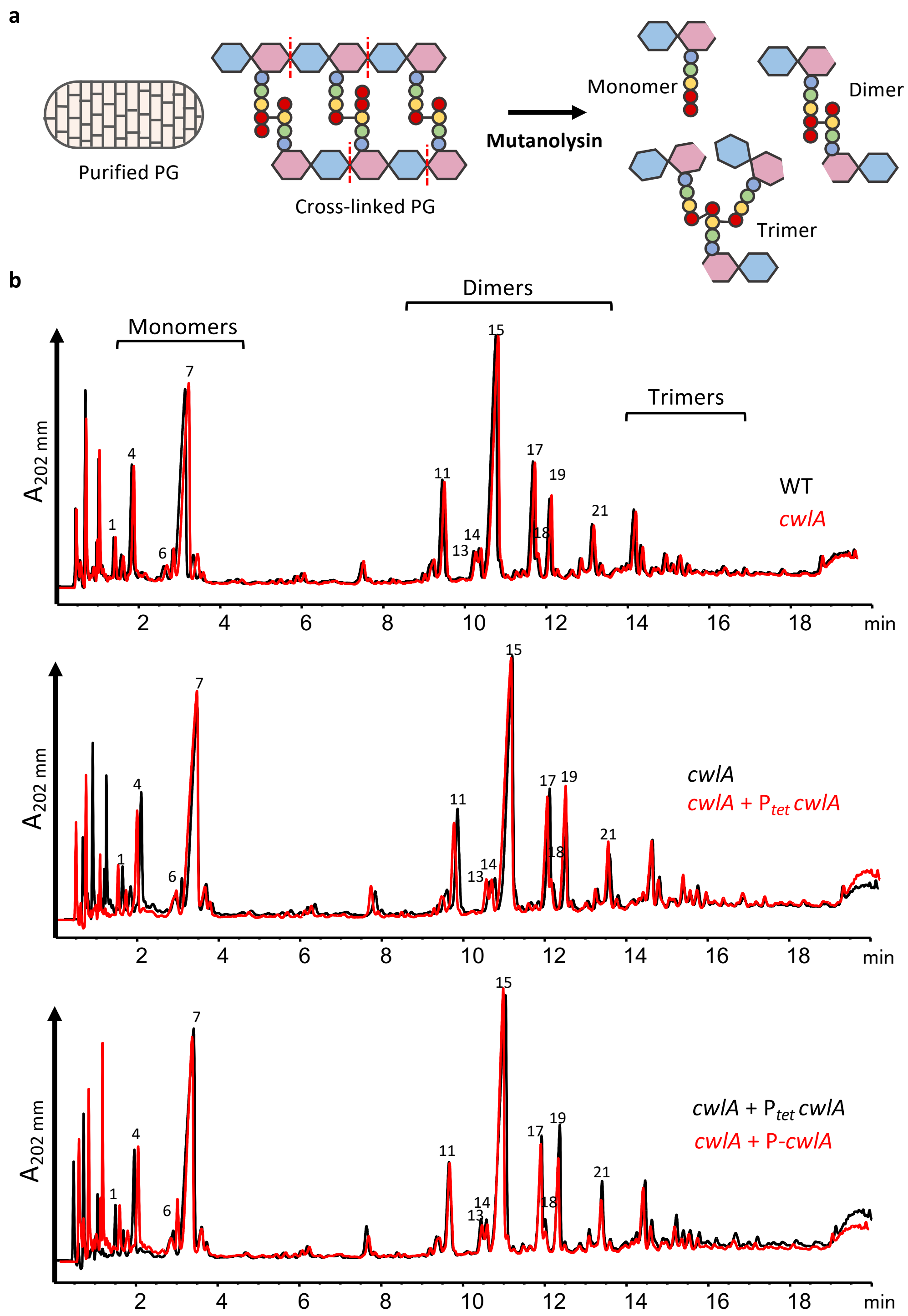

Supplementary Figure 4. Profiles of muropeptides of *cwlA* strains. a, Schematic of peptidoglycan (PG) isolation and digestion for RP-HPLC analysis, mutanolysin treatment of cross-linked PG generate muropeptides species. **b,** RP-HPLC separation profile of muropeptides from WT (P_{tet} *empty*), *cwlA* (P_{tet} *empty*), *cwlA* + P_{tet} *cwlA* and *cwlA* + P-*cwlA* (*cwlA* + pMTL84121-*cwlA*). The profiles were superimposed and the peaks were numbered according to Cuenot et al., 2019 (29). Peak numbers 1, 4, 6, 7 corresponds to monomers and peak numbers 11, 13, 14, 15, 17, 18, 19 and 21 corresponds to dimers. The abundance of each molecule is correlated to the area present under the corresponding peak
