# Supplementary material for: Ser/Thr Kinase-Dependent Phosphorylation of the Peptidoglycan Hydrolase CwlA Controls Its Export and Modulates Cell Division in Clostridioides difficile
Source: mBio. 2021 May 18;12(3):e00519-21. doi: 10.1128/mBio.00519-21 (PMC8262956; doi:10.1128/mBio.00519-21)
Supplement: FIG S5 [file mbio.00519-21-sf005.pdf]

Supplementary Figure 5

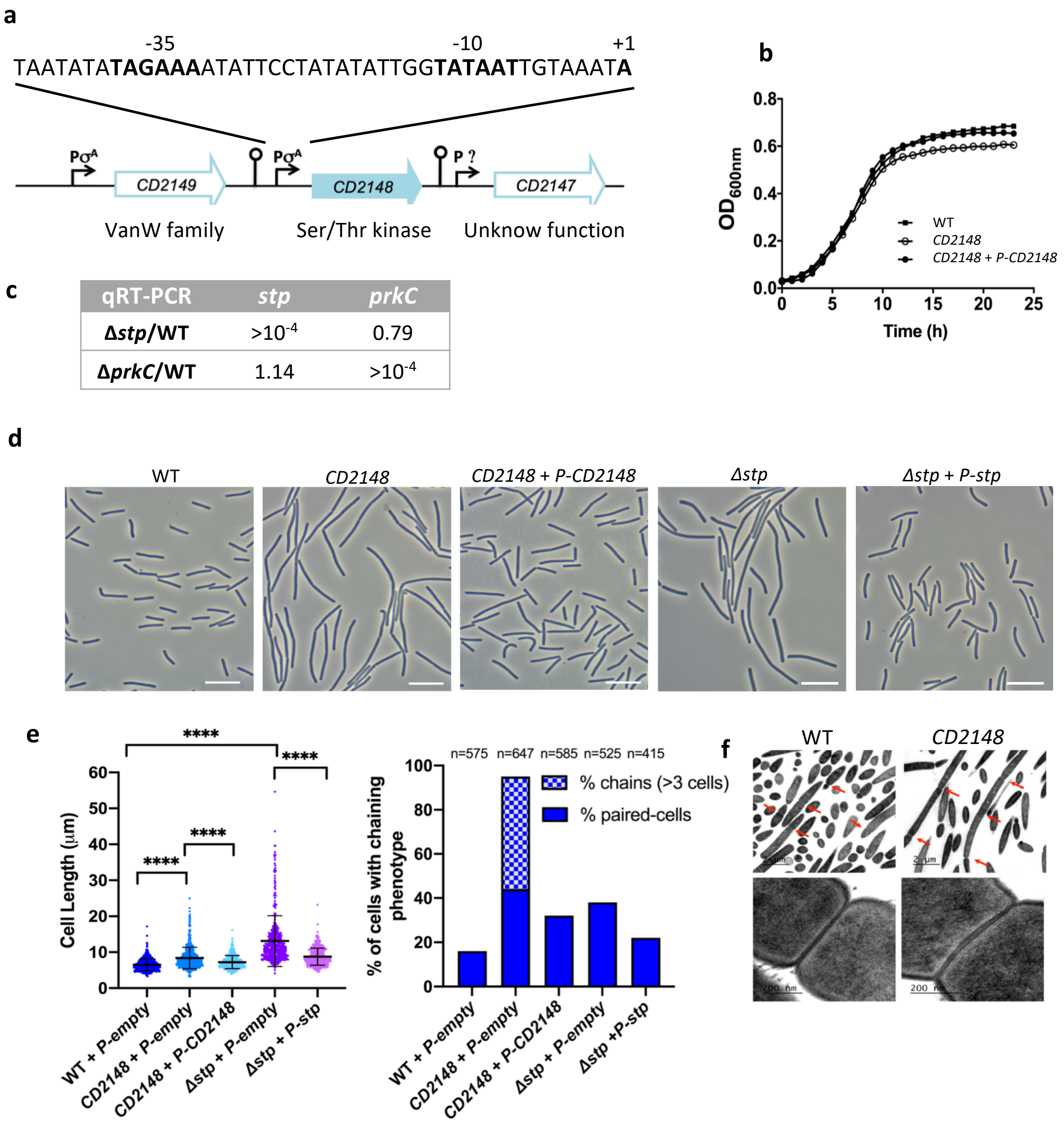

Supplementary Figure 5. Functional characterization of CD2148 and STP. **a**, Genetic organization of the *CD2148* locus. TSS mapping experiment indicated the presence of a σ^A-dependent promoter upstream of *CD2148* and *CD2149* (72). The -35 and -10 boxes as well as the TSS are indicated in bold. **b**, Growth curves of *CD2148* mutant compared to WT strain and the complemented strain *CD2148* + P-*CD2148* in TY. **c**, qRT-PCR analysis of *stp* and *prkC* expression in Δ*stp* and Δ*prkC* mutants compared to the WT strain. The result presented is the mean of the data obtained with 4 independent RNA samples. **d**, Phase contrast images of WT (630Δ*erm* + pMTL84121), *CD2148 (CD2148::erm* + pMTL84121)*, CD2148* + P-*CD2148* (*CD2148::erm* + pMTL84121-*CD2148*), Δ*stp* (Δ*stp* + pDIA6103) and Δ*stp* + P-*stp* (Δ*stp* + pDIA6103-*stp*) cells in TY at exponential phase. **e**, Scatter plots showing cell length (left) and percentage of cells harboring a chaining phenotype (right). *P* values were determined by two-sided Mann–Whitney *U* tests (*****P* < 0.0001); counted 575 (WT + P-*empty*), 647 (*CD2148* + P-*empty*), 584 (*CD2148* + P-*CD2148*), 525 (Δ*stp* + P-*empty*) and 415 *(*Δ*stp* + P-*stp*). **f**, Transmission electron microscopy showing division septa (red arrows, *upper panel*), scale bars 2 µm. Higher magnifications (200 nm) of septa thickness (*lower panel*).
