# Supplementary material for: Ser/Thr Kinase-Dependent Phosphorylation of the Peptidoglycan Hydrolase CwlA Controls Its Export and Modulates Cell Division in Clostridioides difficile
Source: mBio. 2021 May 18;12(3):e00519-21. doi: 10.1128/mBio.00519-21 (PMC8262956; doi:10.1128/mBio.00519-21)
Supplement: FIG S6 [file mbio.00519-21-sf006.pdf]

Supplementary Figure 6

a

	WT		ΔprkC		Δstp		CD2148		Δdouble	
	Site	LP	Site	LP	Site	LP	Site	LP	Site	LP
Phosphosite	**S-136**<br>T-405	1.00<br>1.00	**S-136**	1.00	**S-136**<br>S-229<br>T-405	1.00<br>0.99<br>1.00	S-40<br>**S-136**<br>S-227<br>S-229<br>S-401<br>T-405	1.00<br>1.00<br>0.76<br>0.99<br>0.95<br>1.00	S-40<br>**S-136**<br>S-229<br>S-401	1.00<br>1.00<br>0.99<br>0.95
3D Mapping	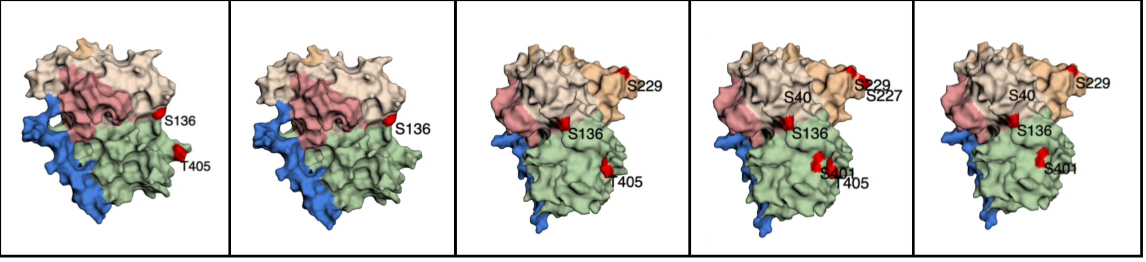									

b

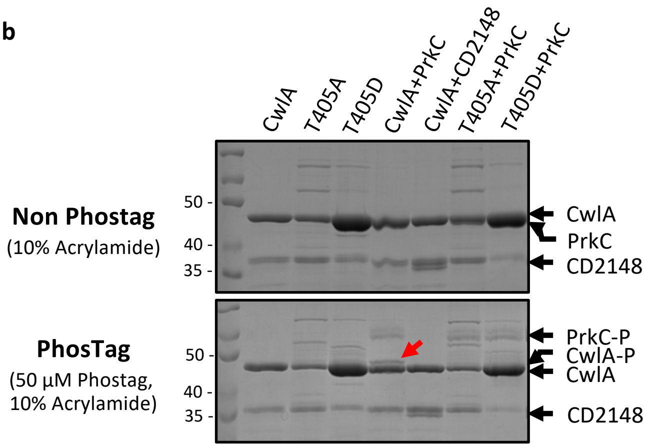

Supplementary Figure 6. *In vivo* and *in vitro* phosphorylation of CwlA. a, Surface representation of CwlA showing phosphorylated residues identified *in vivo* in the STKs/STP mutants with a good localization probability (LP>0.75). Phosphosites S-136 and T-405 are highlighted in black and green, respectively. CwlA is colored by domains (blue, signal peptide; pink, SH3_3.1; light pink, SH3_3.2; orange, SH3_3.3 and green, NlpC/P60) and phosphosites are labeled in red. **b,** *In vitro* phosphorylation assay of CwlA, CwlA-T405A and CwlA-T405D by PrkC or CD2148 and visualized by Phos-tag™ Acrylamide. Red arrow indicates phosphorylated CwlA.
