# Supplementary material for: Ser/Thr Kinase-Dependent Phosphorylation of the Peptidoglycan Hydrolase CwlA Controls Its Export and Modulates Cell Division in Clostridioides difficile
Source: mBio. 2021 May 18;12(3):e00519-21. doi: 10.1128/mBio.00519-21 (PMC8262956; doi:10.1128/mBio.00519-21)
Supplement: FIG S7 [file mbio.00519-21-sf007.pdf]

Supplementary Figure 7

a

qRT-PCR	*cwlA*
**T3-20 ng.mL^{-1} ATc/WT**	1.42
**T3-50 ng.mL^{-1} ATc/WT**	2.23

b

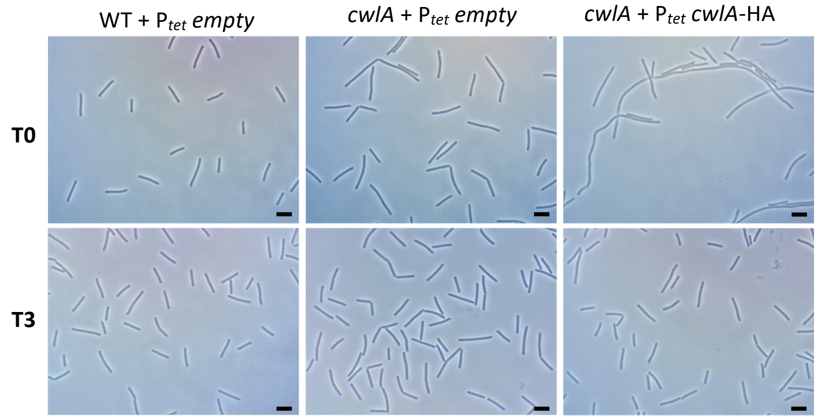

c

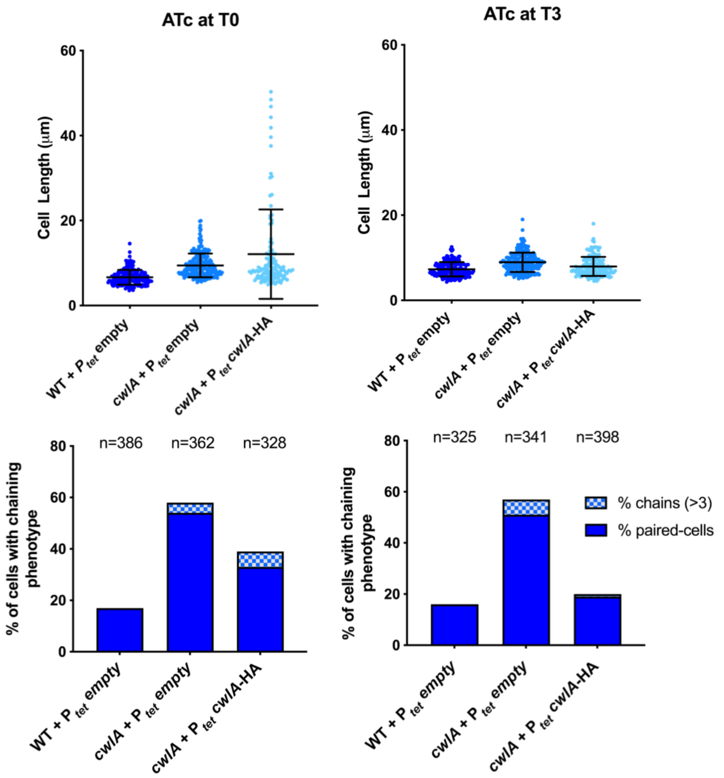

Supplementary Figure 7. Complementation of *cwlA* mutant with pDIA6103-P$_{tet}$ *cwlA*-HA plasmid. a, qRT-PCR analysis estimate the level of *cwlA* expression in the *cwlA::erm* mutant and the complemented strains compared to the WT strain. T3-20 means 3 h of growth followed by 2 h of induction at 20 ng/mL and and T3-50 means 3 h of growth followed by 2 h of induction at 50 ng/mL. The result presented is the mean of the data obtained with 4 independent RNA samples. **b,** Phase contrast images of WT, *cwlA* and *cwlA*+ P$_{tet}$ *cwlA*-HA cells after 5 h of growth in TY. Induction performed with 50 ng/mL ATc from the beginning of inoculation (T0) or after 3 h of growth (T3). Scare bar, 5 µm. **c**, Scatter plots showing cell length with the median and SD of each distribution indicated by a black line (*upper*) and percentage of cells harboring a chaining phenotype (*lower*), n indicates the number of cells counted per strain in a single representative experiment. The images and n values are representatives of experiments performed in triplicate.
