# Supplementary material for: Ser/Thr Kinase-Dependent Phosphorylation of the Peptidoglycan Hydrolase CwlA Controls Its Export and Modulates Cell Division in Clostridioides difficile
Source: mBio. 2021 May 18;12(3):e00519-21. doi: 10.1128/mBio.00519-21 (PMC8262956; doi:10.1128/mBio.00519-21)
Supplement: FIG S8 [file mbio.00519-21-sf008.pdf]

Supplementary Figure 8

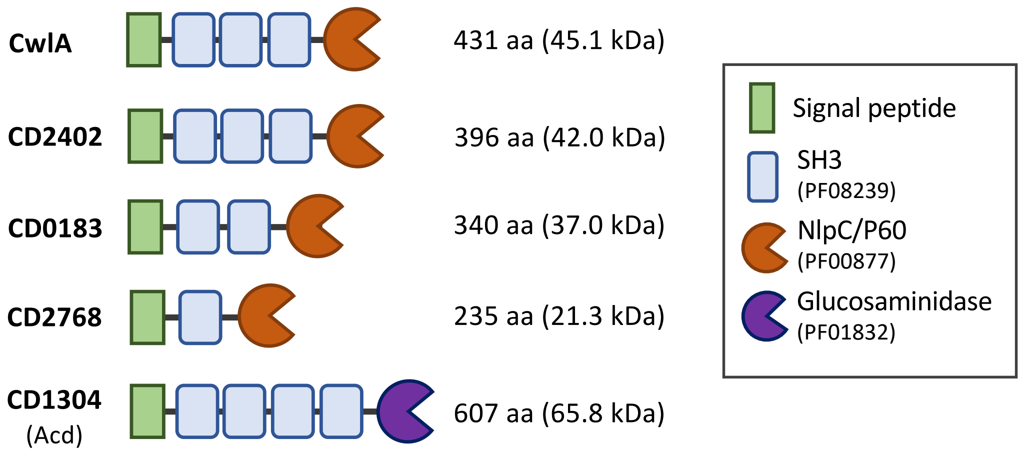

Supplementary Figure 8. Predicted PG-degrading enzymes associated to SH3 domains identified in *C. difficile* 630. Schematic representation of PG-degrading enzymes containing different numbers of SH3 domains (PF08239).
