# Supplementary material for: Ser/Thr Kinase-Dependent Phosphorylation of the Peptidoglycan Hydrolase CwlA Controls Its Export and Modulates Cell Division in Clostridioides difficile
Source: mBio. 2021 May 18;12(3):e00519-21. doi: 10.1128/mBio.00519-21 (PMC8262956; doi:10.1128/mBio.00519-21)
Supplement: TABLE S1 [file mbio.00519-21-st001.docx]

**Supplementary Tables**

**Supplementary Table 1.** Strains and plasmids used in this study

|  | ***E. coli* strains** |  |
| --- | --- | --- |
| NEB 10-beta | derivative of the popular DH10B | NEB |
| HB101 (RP4) | *supE*44 *aa*14 *galK*2 *lacY*1 ∆ (*gpt-proA*) 62 *rpsL*20 (Str^R^)*xyl-5 mtl-1 recA*13 ∆ (*mcrC-mrr*) *hsdS*_B_(r_B_^-^m_B_) RP4 (Tra^+^ IncP Ap^R^ Km^R^ Tc^R^) | Laboratory stock |
| BL21 | F^−^ompT gal dcm lon hsdS_B_(r_B_^-^ m_B_^-^) λ(DE3 [lacI lacUV5-T7 gene 1 ind1 sam7 nin5]) | Novagen |
| M15pRep4 | F^−^, *Φ80*Δ*lacM15*, *thi, lac^-^, mtl^-^, recA*^+^, Km^R^ | Laboratory stock |
|  | ***C. difficile* strains** |  |
| 630∆*erm* | wild-type | Laboratory stock |
| CDIP631 | 630∆*erm* ∆*prkC* | Cuenot *et al.,* 2019 |
| CDIP823 | 630∆*erm* ∆*stp* | This work |
| CDIP1107 | 630∆*erm* ∆*stp* pDIA6103 P_tet_-*stp* | This work |
| CDIP736 | 630∆*erm CD2148::erm* | This work |
| CDIP738 | 630∆*erm* ∆*prkC CD2148::erm* | This work |
| CDIP1150 | 630∆*erm cwlA::erm* | This work |
| CDIP892 | 630∆*erm* (pMTL84121) | Laboratory stock |
| CDIP1735 | 630∆*erm cwlA::erm* (pMTL84121) | This work |
| CDIP893 | 630∆*erm CD2148::erm* (pMTL84121) | This work |
| CDIP1665 | 630∆*erm cwlA::erm* pMTL84121-*cwlA* | This work |
| CDIP894 | 630∆*erm CD2148::erm* pMTL84121-*CD2148* | This work |
| CDIP219 | 630∆*erm* (pDIA6103) | Laboratory stock |
| CDIP1548 | 630∆*erm cwlA::erm* (pDIA6103) | This work |
| CDIP1549 | 630∆*erm cwlA::erm* pDIA6103 P_tet_-*cwlA* | This work |
| CDIP1643 | 630∆*erm cwlA::erm* pDIA6103 P_tet_-*cwlA*-HA | This work |
| CDIP1644 | 630∆*erm cwlA::erm* pDIA6103 P_tet_ -*cwlA* T405A-HA | This work |
| CDIP1645 | 630∆*erm cwlA::erm* pDIA6103 P_tet_ -*cwlA* T405D-HA | This work |
| CDIP1474 | 630∆*erm* pDIA6103 P_tet_ -*cwlA*-HA | This work |
| CDIP1475 | 630∆*erm* ∆*prkC* pDIA6103 P_tet_-*cwlA*-HA | This work |
| CDIP1476 | 630∆*erm CD2148::erm* pDIA6103 P_tet_ -*cwlA*-HA | This work |
| CDIP1477 | 630∆*erm* ∆*stp* pDIA6103 P*_tet_* -*cwlA*-HA | This work |
| CDIP1646 | 630∆*erm* ∆*prkC CD2148::erm* pDIA6103 P*_tet_*-*cwlA*-HA | This work |
| CDIP1659 | 630∆*erm CD2148::erm* P*_tet_*-SNAP*^Cd^*-*CD2148* | This work |
| CDIP1357 | 630∆*erm* P*_tet_*-SNAP*^Cd^-prkC* | Cuenot *et al.,* 2019 |
| CDIP1806 | 630∆*erm cwlA::erm* P-*cwlA-*SNAP*^Cd^* | This work |
| CDIP1699 | 630∆*erm cwlA*::*erm* pDIA6103 P*_tet_*-SH3_3 | This work |
| CDIP1788 | 630∆*erm cwlA*::*erm* pDIA6103 P*_tet_*-NlpC | This work |
|  | **Plasmids** |  |
| pMTL007 | group II intron, ErmBtdRAM2 and ltrA ORF from pMTL20lacZTTErmBtdRAM2 Cm^R^ | Heap *et al.,* 2007 |
| pMTL007::cwlA | pMTL007::*cwlA-*1164s | This work |
| pDIA6485 | pMTL007::*CD2148-*302a | This work |
| pDIA6464 | pMTLSC7315 ∆*stp* | This work |
| pMTL84121 | Clostridia modular plasmid; catP (Cm^R^/Tm^R^) | Heap *et al.,* 2009 |
| pDIA6103 | pRPF185 Δ*gusA* | Soutourina *et al*., 2013 |
| pDIA6912 | pMTL84121-*cwlA* | This work |
| pDIA6712 | pMTL84121-*CD2148* | This work |
| pDIA6928 | pDIA6103-P_tet_-*cwlA* | This work |
| pDIA6935 | pDIA6103-P_tet_-*cwlA*-HA | This work |
| pDIA6991 | pDIA6103-P_tet_-*cwlA* -T405A (phospho-ablative) | This work |
| pDIA6993 | pDIA6103-P_tet_-*cwlA*-T405D (phospho-mimetic) | This work |
| pDIA7026 | pDIA6103-P_tet_-*cwlA-*T405A-HA (phospho-ablative) | This work |
| pDIA7027 | pDIA6103-P_tet_-*cwlA-*T405D-HA (phospho-mimetic) | This work |
| pDIA7047 | pDIA6103-P_tet_-*cwlA*-SNAP^Cd^ | This work |
| pDIA7046 | pDIA6103-P_tet_-SNAP^Cd^-*CD2148* | This work |
| pDIA6855 | pDIA6103-P_tet_-SNAP^Cd^-*prkC* | Cuenot *et al.,* 2019 |
| pDIA6406 | pQE30-*prkC-*KD (kinase domain) | This work |
| pDIA6407 | pQE30-*CD2148-*KD (kinase domain) | This work |
| pDIA7091 | pQE30-*cwlA* | This work |
| pDIA7018 | pQE30-*cwlA-*T405A | This work |
| pDIA7019 | pQE30-*cwlA-*T405D | This work |
| pDIA7128 | pDIA6103 P-*cwlA-*SNAP*^Cd^* | This work |
| pDIA7064 | pDIA6103 P*_tet_*-SH3_3 | This work |
| pDIA7073 | pDIA6103 P*_tet_*-NlpC | This work |

- Cuenot E, Garcia-Garcia T, Douche T, Gorgette O, Courtin P, Denis-Quanquin S, Hoys S, Tremblay Y, Matondo M, Chapot-Chartier MP, Janoir C, Dupuy B, Candela T, Martin-Verstraete I. 2019. The Ser/Thr Kinase PrkC Participates in Cell Wall Homeostasis and Antimicrobial Resistance in Clostridium difficile. *Infection and immunity*, *87*(8), e00005-19. <https://doi.org/10.1128/IAI.00005-19>
- Heap JT, Pennington OJ, Cartman ST, Carter GP, Minton NP. 2007. The ClosTron: a universal gene knock-out system for the genus *Clostridium*. J Microbiol Methods 70:452-464. <https://doi.org/10.1016/j.mimet.2007.05.021>
- Heap JT, Pennington OJ, Cartman ST, Minton NP. 2009. A modular system for Clostridium shuttle plasmids. J Microbiol Methods 78(1):79–85. <https://doi.org/10.1016/j.mimet.2009.05.004>
- Soutourina OA, Monot M, Boudry P, Saujet L, Pichon C, Sismeiro O, Semenova E, Severinov K, Le Bouguenec C, Coppee JY, Dupuy B, Martin-Verstraete I. 2013. Genome-wide identification of regulatory RNAs in the human pathogen *Clostridium difficile*. PLoS Genet 9:e1003493. <https://doi.org/10.1371/journal.pgen.1003493>
