# Supplementary material for: Ser/Thr Kinase-Dependent Phosphorylation of the Peptidoglycan Hydrolase CwlA Controls Its Export and Modulates Cell Division in Clostridioides difficile
Source: mBio. 2021 May 18;12(3):e00519-21. doi: 10.1128/mBio.00519-21 (PMC8262956; doi:10.1128/mBio.00519-21)
Supplement: TABLE S2 [file mbio.00519-21-st002.docx]

**Supplementary Tables**

**Supplementary Table 2.** Oligonucleotides used in this study

| **Primers** | **Sequence (5’→3’)** | **Features** |
| --- | --- | --- |
| IMV988 | AGGCCTGGAGCTCAGATCTG | GA-pDIA6103 |
| IMV989 | GGATCCTATAAGTTTTAATAAAACTTTAA | GA-pDIA6103 |
| IMV993 | CTGGCGTTACCCAACTTAATCG | GA-pMTL84121-Fwd |
| IMV923 | GGGGATCCAAAGGGAGTGTCTCAAAATG | Complementation *CD2148* BamHI |
| IMV924 | CCGCTCGAGAACATAATAACCACTTCCTTTC | Complementation *CD2148* XhoI |
| IMV927 | TGGTCATGAGATTATCAAAAGGGAATAAGGTAGTGAGAGAATG | ACE *CD2579* part I |
| IMV928 | ACTTTTATCACTATAACCGAACCAAATTACTTGTATTTTCTTCTC | ACE *CD2579* part I |
| IMV929 | GTTCGGTTATAGTGATAAAAGT | ACE *CD2579* part II |
| IMV930 | ATCGTAGAAATACGGTGTTTTTTCATCCAAATCAGCTACTTCAA | ACE *CD2579* part I |
| IMV935 | AAGGCCT GAT ATT AGT GGA GCT TGT GG | Complementation *CD2579*-StuI |
| IMV936 | ACGGGATCC CCA ATC TTC CTG ATG ATT TC | Complementation *CD2579*-BamHI |
| IMV937 | AAAAAAGCTTATAATTATCCTTAAATTACCTAGAAGTGCGCCCAGATAGGGTG | *CD2148*-302a-IBS |
| IMV938 | CAGATTGTACAAATGTGGTGATAACAGATAAGTCCTAGAATTTAACTTACCTTTCTTTGT | *CD2148*-302a-EBS1d |
| IMV939 | TGAACGCAAGTTTCTAATTTCGGTTTAATTCCGATAGAGGAAAGTGTCT | *CD2148*-302a-EBS2 |
| IMV941 | GGGAATTCAAAGGGAGTGTCTCAAAATG | Complementation *CD2148* EcoRI |
| EBS universal | CGAAATTAGAAACTTGCGTTCAGTAAAC | Mutant construction |
| IBS cwlA | AAAAAAGCTTATAATTATCCTTAACTAACCATGTAGTGCGCCCAGATAGGGTG | *cwlA* mutant contruction |
| EBS1d cwlA | CAGATTGTACAAATGTGGTGATAACAGATAAGTCCATGTAGGTAACTTACCTTTCTTTGT | *cwlA* mutant contruction |
| EBS2 cwlA | TGAACGCAAGTTTCTAATTTCGATTTTAGTTCGATAGAGGAAAGTGTCT | *cwlA* mutant contruction |
| cwlA F | GGAAGTGCAATTTCTATGGGG | *cwlA* mutant confirmation |
| cwlA R | GTCTGAGTACCACTACAATGG | *cwlA* mutant confirmation |
| ErmRAM F | ACGCGTTATATTGATAAAAATAATAATAGTGGG | Mutagenesis confirmation |
| ErmRAM R | ACGCGTGCGACTCATAGAATTATTTCCTCCCG | Mutagenesis confirmation |
| pMTLseq-F | GGGATCCTCTAGAGTCG | intron retargeted sequencing |
| pMTLseq-R | CAGATTCTCGGCATCGC | intron retargeted sequencing |
| TG43 | TCCATGGAGATCTCGAGGCCTACTTCCTAAAAATCACTAATTTTAAG | GA- pMTL84121-Fwd |
| TG44 | CGATTAAGTTGGGTAACGCCAGAGTTCTAATTTTTTAATAAGAAATTGTACT | GA-pMTL84121-Rev |
| TG45 | TATTAAGCGTAATCTGGAACATCATATGGATAAACAAATCTTCTTGCACCAAG | *cwlA*-HA-Rev |
| TG46 | AGTACAATTTCTTATTAAAAAATTAGAA | *cwlA*-HA-Fwd |
| TG73 | GTTAAAGTGGATAATTTAACTAGTTCA | *cwlA* T405-Fwd |
| TG74 | CTTATTAGGATTAGCCTGAGTACC | *cwlA* T405A-Rev |
| TG76 | CTTATTAGGATTATCCTGAGTACC | *cwlA* T405D-Rev |
| TG97 | AACAAATCTTCTTGCACCAAGTAA | *cwlA* SNAP-FI-Rev |
| TG98 | TAAGTACAATTTCTTATTAAAAAATTAGAA | *cwlA* SNAP-FI-Fwd |
| TG99 | TTACTTGGTGCAAGAAGATTTGTTGGATCCGCAGCTGCTGATAAAGATTGTGAAATGAAGAGAACC | *cwlA* SNAP-FII-Fwd |
| TG100 | TTCTAATTTTTTAATAAGAAATTGTACTTATTACCCAAGTCCTGGTTTCCCCA | *cwlA* SNAP-FII-Rev |
| TG93 | AATTTTATAGGCAATAGATATGAGGTA | SNAP *CD2148*-FI-Fwd |
| TG94 | CAACCATGTCACCTCCTCATA | SNAP *CD2148*-FI-Rev |
| TG95 | TATGAGGAGGTGACATGGTTGGATAAAGATTGTGAAATGAAGAGAACC | SNAP *CD2148*-FII-Fwd |
| TG96 | TACCTCATATCTATTGCCTATAAAATTAGCAGCTGCGGATCCCCCAAGTCCTGGTTTCCCCA | SNAP *CD2148*-FII-Rev |
| SAT117 | CGCGGATCCATGGGAGATACAATTTTAGGAAATCG | pQE30-*prkC*, BamHI-Fwd |
| SAT118 | CGGGGTACCTTAATCATCTTCTTCATAAAAATCATC | pQE30-*prkC*, KpnI-Rev |
| SAT119 | CGCGGATCCTTGAATTTTATAGGCAATAG | pQE30-*CD2148*, BamHI-Fwd |
| SAT264 | CGGGGTACCTTAACTTTTATATTCTTTTTTAACTTG | pQE30-*CD2148*, KpnI-Rev |
| SAT285 | GCTAGGATCCCTTGAAAAGGGAACAGTAACAGC | pQE30-*cwlA*, BamHI-Fwd |
| SAT286 | CGGGGTACCCTAAACAAATCTTCTTGCACC | pQE30-*cwlA*, KpnI-Rev |
| TG107 | ACTTGGTCCACTTCTTATATTCAA | *cwlA*-NlpC Rev |
| TG108 | AAAAACCAAATTCATCATCTAACCAA | *cwlA*-NlpC Fwd |
| TG101 | TGGCTTTGAGTTACTATTAGATTG | *cwlA*-SH3_3 Rev |
| TG102 | TAGTAAGTACAATTTCTTATTAAAAAATTAGA | *cwlA*-SH3_3 Fwd |
| IMV906 | GGCAAATGTTGGAGATAGCA | qRT-PCR *CD2579* Fwd |
| IMV907 | TTCAACCACGGAATGGTCTA | qRT-PCR *CD2579* Rev |
| IMV904 | AAAAACAGGAAAGCCCAAAA | qRT-PCR *prkC* Fwd |
| IMV905 | ATTTATTGCCAAACCCTCCA | qRT-PCR *prkC* Rev |
| TG122 | GGATGGGGAAGTGCAAAATAC | qRT-PCR *cwlA* Fwd |
| TG123 | GATTTCCACTAATAGTTGTCCCAC | qRT-PCR *cwlA* Rev |
